# Experimental and numerical study on gas production decline trend under ultralong-production-cycle from shale gas wells

**DOI:** 10.1038/s41598-023-37244-4

**Published:** 2023-07-03

**Authors:** Xianggang Duan, Yingying Xu, Wei Xiong, Zhiming Hu, Shusheng Gao, Jin Chang, Yongsheng Ge

**Affiliations:** 1 PetroChina Petroleum Exploration and Development Research Institute, Beijing, 100083 China; 2 University of Chinese Academy of Sciences, Beijing, 100493 China; 3 The Fourth Oil Production Plant of PetroChina Huabei Oilfield Company, Hebei, 065000 China

**Keywords:** Natural gas, Fluid dynamics

## Abstract

It is of engineering interest to explore recovered shale gas composition and its effects on total gas production trend over a long-term extraction period. However, there are previous experimental studies mostly focused on short term development for small scaled cores, which is less convincing to mimic reservoir-scaled shale production process. In addition, the previous production models mostly failed to account for comprehensive gas nonlinear effects. As a result, in this paper, to illustrate the full-life-cycle production decline phenomenon for shale gas reservoir, dynamic physical simulation was performed for more than 3433 days to simulate shale gas transport out of the formations over a relatively long production period. Moreover, a five-region seepage mathematical model was then developed and was subsequently validated by the experimental results and shale well production data. Our findings show that for physical simulation, both the pressure and production declined steadily at an annual rate of less than 5%, and 67% of the total gas in the core was recovered. These test data supported earlier finding that shale gas is of low flow ability and slow pressure decline in the shale matrices. The production model indicated that free gas accounts for the majority of recovered shale gas at the initial stage. Based on a shale gas well example, free gas extraction makes up 90% of produced total gas. The adsorbed gas constitutes a primary gas source during the later stage. Adsorbed gas contributes more than 50% of the gas produced in the seventh year. The 20-year-cumulative adsorbed gas makes up 21% of the EUR for a single shale gas well. The results of this study can provide a reference for optimizing production systems and adjusting development techniques for shale gas wells throughout the combinations of mathematical modeling and experimental approaches.

## Introduction

China is renowned for its abundant shale gas resources, with total recoverable reserves estimated at 21.84 trillion m^3^. In the southern region of China, recoverable marine facies shale gas resources amount to 14.1 trillion m^3^. Nearly a decade of exploration and development efforts has enabled China to commercialize shale gas development. In 2021, China’s total shale gas production reached 22.8 billion m^3^^1^. Integrated geological and engineering technologies for reservoirs shallower than 3500 m have gradually taken shape, while the primary technologies used to explore and develop shale gas (e.g., horizontal well drilling, staged volume fracturing, and factory-like operation technologies) have basically been established. Nevertheless, there are still some urgent problems to be solved on development technologies and seepage theories for shale gas systems. For example, shale gas production varies appreciably between wells and declines in a yet-to-be-established pattern, while the available shale gas development systems require optimization. These problems, to some extent, hinder the efficient, sustained development of shale gas^2^.

Shale gas reservoirs differ from conventional gas reservoirs in that shale function as both the source rocks that generate natural gas and the reservoir and cap rocks that host and preserve natural gas. The Sichuan Basin and its peripheral areas—China’s current main shale gas-producing region—provide a marine facies deep-water continental-shelf sedimentary environment. Here, high-quality shale reservoirs distributed continuously over a large area have developed in the Lower Silurian Longmaxi Formation (LMX Fm). Characteristically, the shale reservoirs in China differ relatively substantially from those in the United States. Devoid almost completely of highly permeable sandy interlayers and with a relatively low content of total organic carbon (TOC) (2–8%), the shale reservoirs distributed in China are relatively highly thermally mature (vitrinite reflectance in oil: 2.5–4%) and located at great depths (2500–5500 m). Moreover, these reservoirs were frequently reformed by regional tectonic movements during the later stage, resulting in varying fracture development patterns and in situ stresses and even more complex geological conditions for development^3,4^. Shale contain abundant organic matter (OM) with nanoscale organic pores at a porosity generally ranging from 2 to 5%. Gas has low flow ability, usually at the nano- and microdarcy levels, in shale matrices where microfractures are commonly present. To increase the flow conductivity and discharge area of a shale matrix, artificial hydraulic fracturing must be conducted to connect its natural fractures to form a complex fracture network that can provide gas flow channels. Shale gas exists predominantly in the free state in the fractures, pores, and other accumulation spaces in black muddy shales and in the adsorbed state in kerogen and clay particles and on the surfaces of their pores^5,6^. An extensive presence of adsorbed gas is a primary characteristic of shale gas reservoirs that distinguishes them from dense sandstone gas reservoirs. The proportion of adsorbed gas varies relatively significantly between regions, ranges approximately from 20 to 80% in shale gas fields, and has an impact on the corresponding development mode^7–9^.

The extremely low porosity–permeability and complex gas transport mechanisms in a shale reservoir result in a unique L-shaped characteristic production curve that reflects high initial production, followed by a rapid decrease and low but stable production during the later stage^10^. An onsite shale gas production well outputs gas at an irregular rate. In particular, the fracturing fluid flowback and the initial gas production process preclude the conventional methods to estimate its productivity from its pressure and production data. The gas production rate becomes somewhat regular during the middle and later stages of production. However, changes in the production system present challenges for model calculations. For example, opening and closing a well multiple times changes its bottom hole pressure, which in turn leads to discontinuous production data and thus large errors in production decline analysis and estimated ultimate recovery (EUR) estimates. Estimations calculated by empirical or semiempirical methods from pressure and production data alone are often grossly inaccurate^10–13^. Zhang et al.^14^, Gao et al.^15^, Duan et al.^16^, Hu et al.^17^, and Duan et al.^18^ illustrated gas production patterns during shale gas development in depletion mode based on results obtained from physical simulation tests. However, the small cores and short development times used in these studies make their findings less convincing. Therefore, further research efforts are warranted to improve the understanding of full-life-cycle patterns. Due to abundant micropores and microcracks in shale formation, the flow mechanisms in shale reservoir are much more complex than that in the conventional gas reservoir^19,20^. As a consequence, shale gas flow is a complicated combination of all flow regimes, which includes desorption, viscous flow, slip flow, and transition flow^21,22^. The gas viscous flow in conventional reservoir is described by Darcy flow, and the gas slippage is mainly dominated by pressure, temperature and rock pore structure^21^. The temperature and pressure of shale gas reservoirs will exceed the critical temperature (− 82.6 °C) and critical pressure (4.59 MPa) of methane. It is therefore obvious to be shown that gas adsorption process in the shale matrix is described as supercritical adsorption, which is different from Langmuir adsorption. The conventional Langmuir adsorption equation is not suitable for characterizing the high-pressure isothermal adsorption curve for shale reserviors. Based on the pore length scale difference of shale storage space, several supercritical adsorption models are proposed, which aids to explain the shale gas adsorption mechanism reasonably. The supercritical adsorption models have been generally classified as four modes: excess adsorption model^23^, supercritical adsorption model based on the potential theory^24^ and SLD-PR theoretical model^25^. As a result, instead of characterizing gas flow based on conventional Darcy flow, it is more reliable to incorporate multi nonlinear flow regimes and model the gas dynamics in organic nanopores using different methods, such as the multi-continuum, molecular dynamics, Monte Carlo and the lattice Boltzmann method (LBM), in organic nanopores^26,27^. The unconventional reservoir production cannot be directly derived from production calculations applied in conventional formations. Wu et al.^28^, Ahmadi et al.^29^, Wu et al.^30^, Zhang et al.^31^, Zhu et al.^32^, Zhao et al.^33^, and Li et al.^34^ investigated coupled flow and production prediction models for multistage-fractured horizontal shale gas wells. Different models focus on different aspects and have their own advantages and disadvantages. Although most of these models consider the effects of the adsorption, desorption, and diffusion of shale gas on seepage, they only examine single influencing factors and fail to comprehensively account for the nonlinear effects of several aspects of the gas (e.g., its physical properties at high pressures, supercritical desorption characteristics, and multiple flow mechanisms) during the gas production process on gas-well production patterns. Theoretical models also lack support from test data.

**Figure 1:**
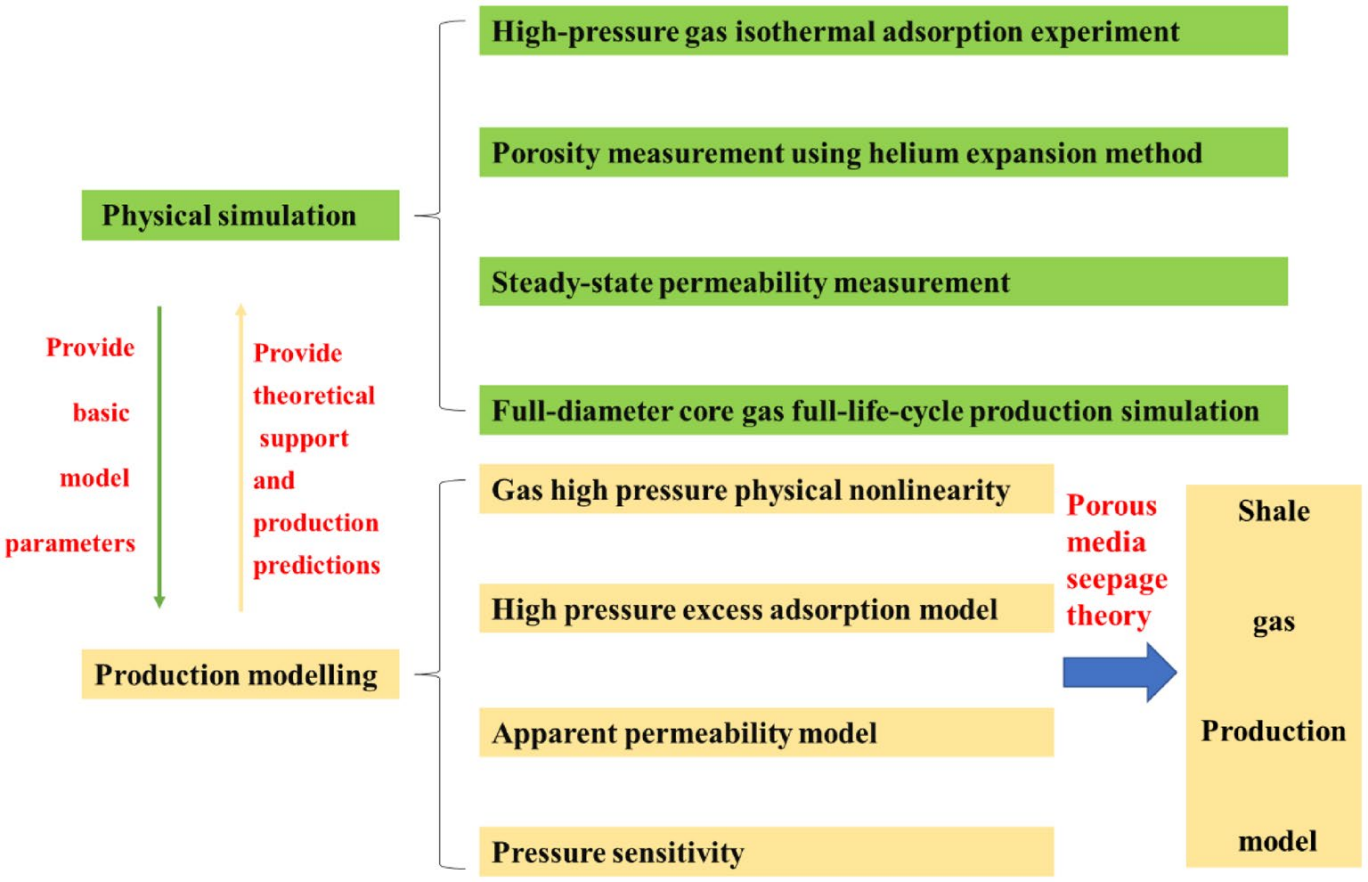
Flowchart of physical simulations and production model implemented in the study.

Hence, to address the problems encountered during shale gas development (e.g., discontinuous production data, short single-well production times, and production estimation difficulties), it is necessary to establish physical and numerical methods to dynamically simulate shale gas development, investigate the gas transport mechanisms in shale reservoirs and the physical processes involved in gas production, and improve the methods used to evaluate shale gas reservoirs and optimize their production, illustrated in Fig. 1. This study examined the full-life-cycle dynamic production of shale gas wells. Considering the unique physical characteristics of shale reservoirs, a full-diameter shale core was retrieved from a shale reservoir currently under extractions for analysis. Then, the original conditions of the core (including parameters such as the original pressure, water content, gas content, and adsorbed gas proportion) at the in situ temperature and pressure in the reservoir were determined. Subsequently, a shale gas development process in depletion mode was simulated under production conditions consistent with those in the actual gas well. The obtained pressure and production data were consistent with and can be used to reasonably and effectively explain the characteristic production curves of the gas well. A five-region productivity model that accounts for matrix adsorption at high pressures and the fractures stress sensitivity was established and subsequently validated based on the test data. Afterwards, a single-well model was developed to analyze the full-life-cycle dynamic production characteristics of a shale gas well and the adsorbed and free gas proportions during production period. The results of this study can provide important theoretical and practical guidance for efficiently developing shale gas wells.

## Physical simulation experiment method

A full-diameter core sample of the Longyi_1_ sublayer of the LMX Formation retrieved from well Y10 in Zhaotong in southern Sichuan, with the collection depth of 2450 m, was used in this test. The length and diameter measurements of the core were 15 cm and 10 cm, respectively. To maximally preserve its original characteristics, the core was quickly cleaned and then placed in a pressure-retaining coring device immediately after retrieval from the borehole while on site, followed by saturation with natural gas to 10 MPa. The valves at both ends of the core were then closed before it was transported to the laboratory for analysis. A parallel sample design was used in the test. Samples retrieved from the areas adjacent to the area where the full-diameter core was collected were tested to determine the physical properties of the shale and were also used for comparison (see Table 1 for the results). For example, some samples were ground to powder that could pass through sieves with mesh sizes of 60–100. The powder was subsequently subjected to an isothermal adsorption test to determine the adsorbed gas content. Because the shale in the study area contains developed beds, core plugs were prepared using the wire-cutting technique to inhibit microfracture initiation during the conventional string drilling process. Methane (purity: 99.99%) was used in the test to simulate the shale gas flow in the reservoir. A GAI-100 high-pressure gas adsorption isotherm system (Core Laboratories, the United States) was used to measure the adsorbed gas content. The helium expansion method was adopted to measure the porosity on a PoroPerm-200 pore permeability measurement system. The classical steady-state permeability measurement method was employed in tandem with Klinkenberg’s theory (included to account for the slip effect) to calculate the corresponding Klinkenberg permeability. Table 1 summarizes the basic parameters of the core used in the test. Evidently, both the porosity and permeability of the shale in the reservoir are very low, averaging approximately 2% and 0.00005 mD, respectively.

**Table 1:** Basic physical property parameters of the core.

Core No	Length (cm)	Diameter (cm)	Mass (g)	Porosity (%)	Permeability (mD)	TOC content (%)	Specific surfaces area (m^2^/g)	Gas content (m^3^/t)	Adsorbed gas content (m^3^/t)	Free gas content (m^3^/t)
Y10-1	15	10.05	3044.6	2.03	0.00005	3.2	12.81	2.2	0.54	1.66

**Figure 2:**
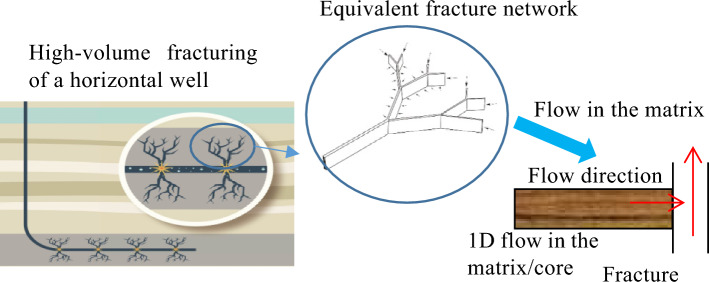
Schematic diagram of the 1D flow of the gas provided by a matrix toward the fractures.

**Figure 3:**
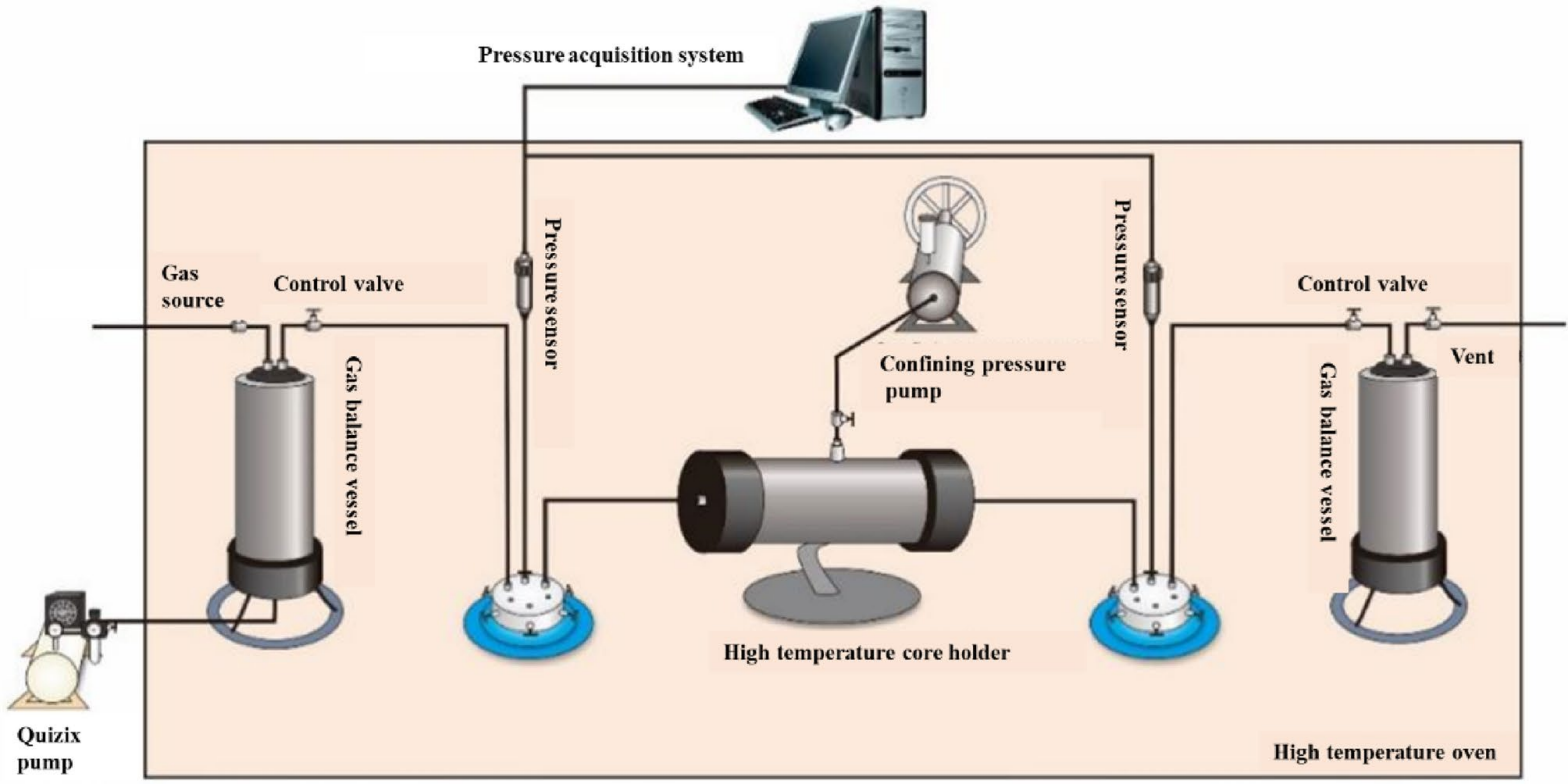
Schematic diagram of dynamic shale gas development simulation experiment setup.

With a flow conductivity on the order of 100 mD m (considerably higher than the permeability of the matrix), artificial fractures in a shale gas reservoir can be regarded as infinite conduction fractures. Therefore, the gas provided by the matrix can be considered to flow in a one-dimensional (1D) manner from the matrix to the nearby fractures (Fig. 2). A method was established to physically simulate 1D gas flow based on the core collected in this study from the reservoir. This method was subsequently used to examine the capacity of the matrix to provide gas to the fractures at the formation pressure on a test setup developed in-house to physically simulate the coupled desorption, diffusion, and seepage of shale gas (Fig. 3). The main components of the setup included a Teledyne ISCO pump, a high-pressure core holder, intermediate containers, a confining pressure pump, a gas mass flowmeter, and high-precision pressure sensors. Figure 3 shows the test procedure described in detail as follows. (1) The core holder containing the core retrieved from the field was connected to the simulation test setup. The high-pressure pump was used to increase the natural gas pressure in the high-pressure intermediate containers to 28 MPa and maintain it at this level. The core was saturated again and pressurized to the formation pressure level (28 MPa) through injection of methane at both ends, while the confining pressure was simultaneously increased to 50 MPa. As a corollary of the extremely high density and adsorption behavior of the shale, fully restoring the core to its in situ state required a very long time. The pressurization and saturation process continued for 200 days and was terminated when the pressures in the intermediate containers at the inlet and outlet of the core holder no longer decreased. Under this condition, the shale gas was almost completely restored to its original state in the host formation. Subsequently, the inlet and outlet control valves (valves 1 and 2, respectively) were closed, followed by the removal of the high-pressure gas source. Thus, the setup was ready for a full-life-cycle production simulation test. (2) Valve 2 was opened to commence the test. To prevent an excess gas flow rate at the initial stage and maintain a reasonable back pressure at the outlet, the back pressure was adjusted to the atmospheric pressure when the flow rate fell within a suitable range. Under this condition, full-life-cycle long-term gas production from the shale gas well was dynamically simulated. (3) Throughout the simulation test, the inlet and outlet pressures and the outlet flow rate were continuously measured in real time using the inlet and outlet pressure sensors (sensors 1 and 2, respectively) and the outlet gas mass flowmeter, respectively, and the data were collected using a computer data acquisition system. To improve the test accuracy, the gas flow was measured using the inlet gas mass flowmeter during the initial stage when the flow rate was relatively high, combined with use of the bubble or micropipe drainage method during the low-production stage.

## Physical simulation test results and discussion

### Isothermal adsorption results and pore structure characterization

**Figure 4:**
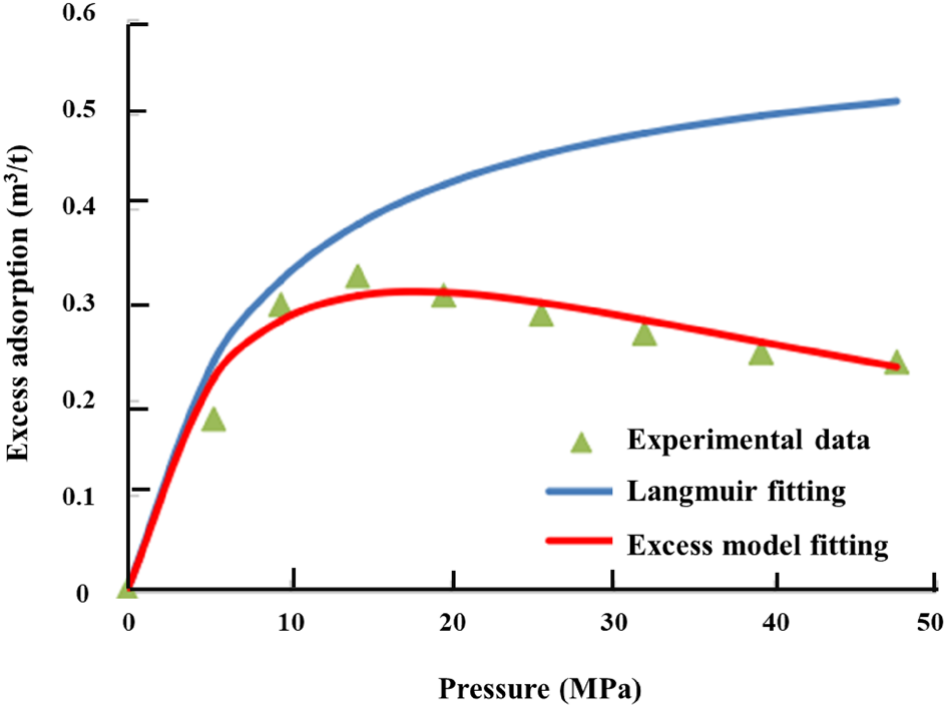
Measured excess adsorption curve and model fits.

Figure 4 shows the isothermal adsorption curve experimentally obtained by testing dry samples using the adsorption isotherm system. As is presented in the Fig. 3, the excess adsorption curve is obtained based on the high pressure absorption characterization model^18^, written in the Eq. (1):

1 $${V}_{a}={V}_{\mathrm{L}}\frac{p}{{p}_{L}+p}\left(1-\upbeta {\rho }_{g}\right)$$

where ${V}_{a}$ is excess gas adsorption volume m^3^/kg; V_L_ is Langmuir volume, m^3^/kg; P_L_ is Langmuir pressure, Pa; $\upbeta$ is modification factor caused by high pressure and high temperature adsorption, m^3^/kg; P is gas pressure in seepage field, Pa; ${\rho }_{g}$ is gas density, kg/m^3^.

Observation of the measured isothermal adsorption curve in Fig. 4 shows the following. The adsorption (i.e., excess adsorption) increased sharply as the pressure increased before reaching 10 MPa. As the pressure increased from 10 to 15 MPa, the excess adsorption increased slowly (a decrease in the excess adsorption was observed in some samples). Further increasing the pressure beyond 15 MPa reduced the excess adsorption, as observed in all the samples. This finding is consistent with that obtained from a high-pressure supercritical isothermal adsorption test. The typical pattern of the high-pressure supercritical isothermal adsorption curve of the shale differs from the monotonic increase pattern of its low-pressure isothermal adsorption curve, suggesting that the excess adsorption was measured, instead of the absolute adsorption (i.e., the actual adsorption). An absolute adsorption correction model was used to correct the excess adsorption curve. An absolute adsorption curve (the red curve in Fig. 4) was thus obtained. The high-pressure isothermal adsorption pattern of the shale, characterized by an increase and a subsequent decrease in the excess adsorption with an increase in the pressure, is difficult to describe with the Langmuir model (used to describe absolute adsorption) and other subcritical models^29^. Therefore, a new model must be established. Observation of Eqs. (2) reveals the following relationship between excess and absolute adsorption:

2 $$G_{ex} = G_{abs} \left( {1 - \frac{{\rho_{g} }}{{\rho_{a} }}} \right)$$

where $G_{ex}$ is the excess absorbed gas accumulated in the shale rock, m^3^; $G_{abs}$ is amount of adsorbed gas accumulated in the shale formation, m^3^; $\rho_{g}$ is free gas density, kg/m^3^; $\rho_{a}$ is absorbed gas density, kg/m^3^.

An assumed adsorbed-phase density is required to use the equation presented above. Researchers have fitted excess adsorption curves with the liquid-phase density (423 kg/m^3^), van der Waals density (373 kg/m^3^), and critical density as the adsorbed-phase density^18,30^. In this study, a revised Langmuir model was used to calculate the adsorbed gas content.

### Pore structure characteristics

**Figure 5:**
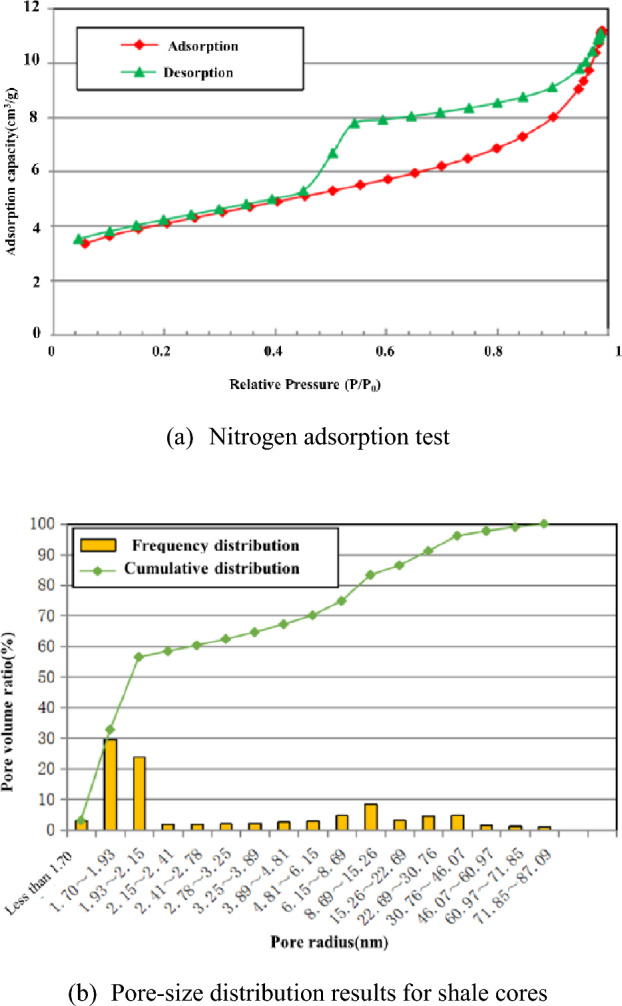
Nitrogen adsorption test (**a**) and pore-size distribution results (**b**) for the samples.

A nitrogen adsorption analyzer was used to generate a nitrogen adsorption curve for the samples, as shown in Fig. 5. Evidently, the adsorption–desorption curve is not prominent. Observation of the hysteresis loop reveals that the adsorption increased rapidly as the relative pressure approached 1. The hysteresis loop also appears relatively small. These findings mainly reflect the presence of parallel plate pores with four open sides. Moreover, the shape of the hysteresis loop conforms to that of a Type H4 hysteresis loop, indicating the presence of slit pores. Relatively well-developed pores in various diameter ranges (from micropores to large pores) were all observed, and they were relatively well interconnected. Analysis of the test results for the samples reveals a specific surface area of 12.81 m^2^/g, a Barrett–Joyner–Halenda (BJH) total pore volume of 0.0276 mL/g, and an average pore diameter of 9.38 nm.

Focused ion beam (FIB) is a technique used to achieve microbeam fabrication with an ion beam focused to a sub micrometer- or even nanometer-sized spot through a deflection system. Scanning electron microscopy (SEM) is employed to produce images of a sample. In this technique, various types of signals (e.g., secondary and backscattered electron signals) are first generated from the sample through the interactions between high-energy electrons and matter and are subsequently converted sequentially and proportionally through a detector into video signals that are then amplified and adjusted for point brilliance to form an image. In this study, a Helios NanoLab 650 dual beam SEM system (ion beam voltage: 500 V–30 kV; electron beam voltage: 350 V–30 kV) was used to produce two-dimensional microimages of the core samples, with the goal of revealing the pore structure (laminae and microfractures), nanoscale particles and pores, and mineral composition on the sample surfaces.

**Figure 6:**
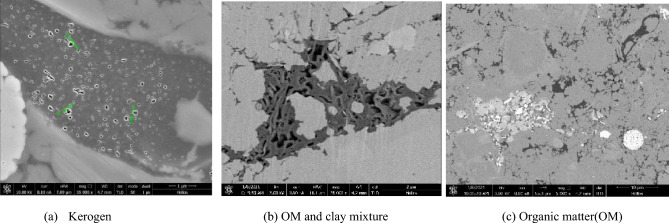
FIB-SEM images of the samples.

Inspection of the SEM images shows an extensive presence of pores in the OM. As seen in Fig. 6a, a large number of circular or oval-shaped bubble pores of a relatively uniform size were present in the kerogen. Most of these pores exhibited a spongy or honeycomb-like appearance with a diameter less than 100 nm. Figure 6b shows organic pores in a mixture of OM and clay. Changes in geological conditions during accumulation and thermal evolution lead to the development of multitudinous micropores or microcracks in OM. Distributed primarily along microbedding planes or sedimentary discontinuities, OM tends to form interconnected pore networks and thus has a relatively high permeability. Banded OM, pyrite, and a large amount of quartz and calcite are visible in Fig. 6c. The pores in this sample developed predominantly within the OM and were relatively poorly connected, conforming to the low permeability of the matrix.

### Production pressure and production decline curves

**Figure 7:**
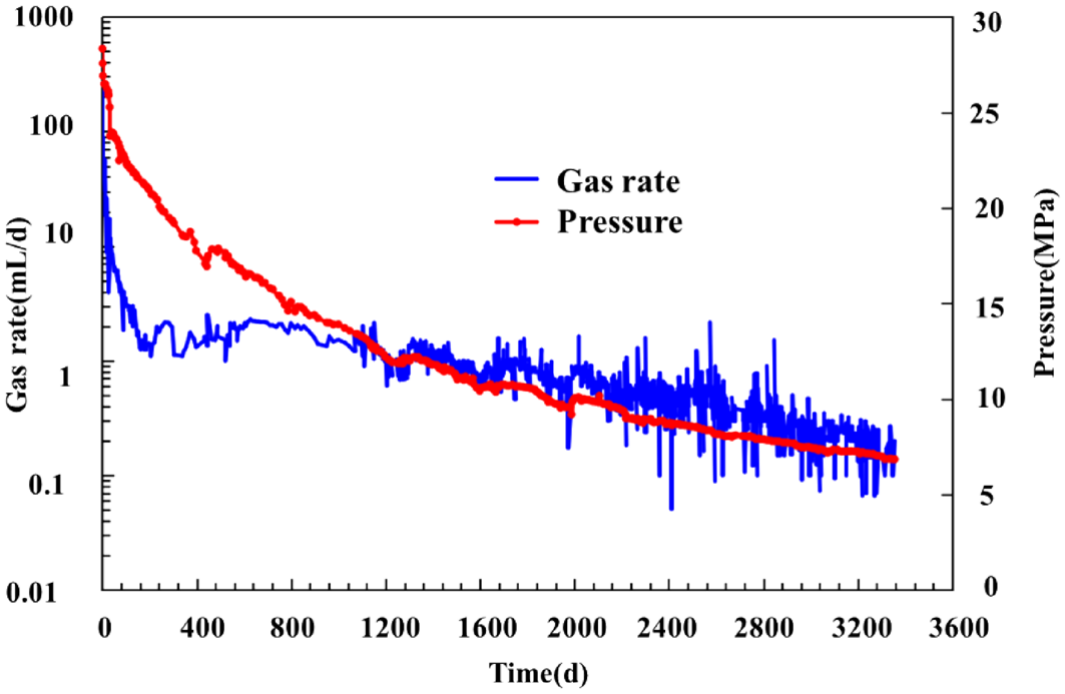
Measured inlet pressure and production decline rate curves of the full-diameter core.

Figure 7 shows the inlet pressure and gas production rate curves obtained from the test after 3356 days (September 15, 2012, through November 30, 2021).

**Figure 8:**
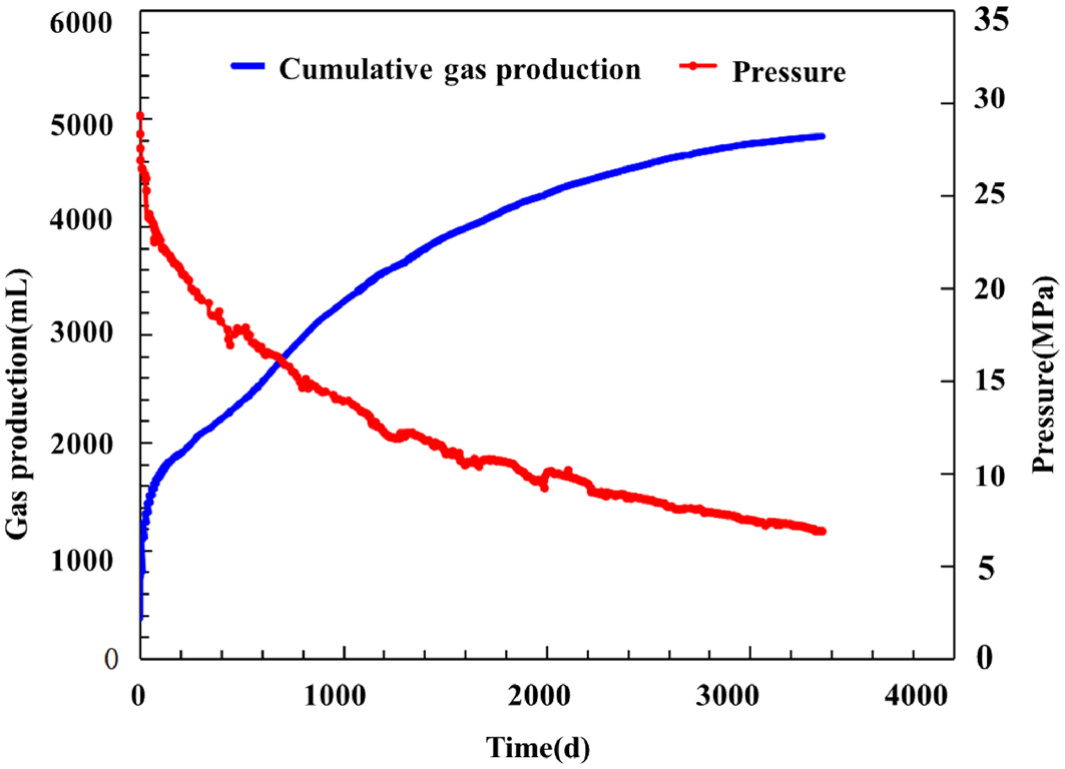
Measured cumulative production and gas production decline rate curves of the full-diameter core.

Analysis of the production curve in Fig. 7 and Fig. 8 reveals the following. The tested core produced gas at a rate that was high but decreased rapidly during the initial stage and subsequently quickly stabilized then remained stable for a protracted period of time. The production decline curve closely resembles a single-well production decline curve. Gas was produced at a high rate during the first 40 days. Throughout this stage, the pressure decreased from 28 to 20 MPa, while the gas production rate decreased rapidly from 140 to less than 10 mL/d. Subsequently, there was a decrease in both the rates at which the daily gas production decreased and the cumulative production increased. The pressure and daily gas production decreased to 18 MPa and 2 mL/d, respectively, after 200 days of production, followed by a long-lasting stage of low but stable production, during which the gas production rate remained stable and decreased extremely slowly. By the end of the production period examined in this study (i.e., the 3356th day), the inlet pressure and daily gas production had stabilized at 6.8 MPa and approximately 0.2 mL, respectively, while the cumulative gas production had reached 4837 mL. According to the calculation of the gas content, the core contained 6710 mL of gas in total. Taken together, these results indicate that 72.1% of the gas had been recovered. The extremely low permeability and adsorption and desorption behaviors were the primary causes of the slow pressure decline and a long period of low but stable gas production observed in the shale.

**Figure 9:**
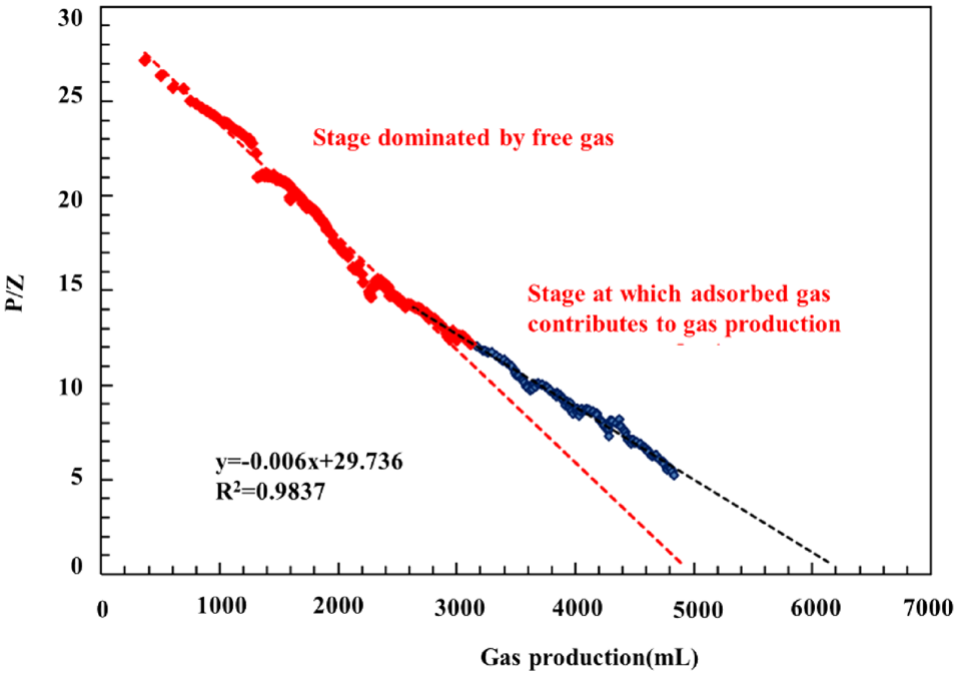
Measured relationship between the cumulative gas production and P/z of the full-diameter core.

Figure 9 shows a plot of the apparent pressure versus the cumulative gas production. Observation of Fig. 9 shows a relatively notable linear relationship between the cumulative gas production and apparent pressure with a decrease the apparent pressure (as indicated by the red dots in Fig. 9). The apparent formation pressure decreased further below 12 MPa at a decreasing rate, as indicated by the deviation from the blue straight line in Fig. 9, the extent of which increased as the apparent formation pressure decreased. This finding suggests a critical desorption pressure of 12 MPa for the adsorbed gas in the shale core at room temperature, which is consistent with that obtained from a high-pressure adsorption test on shale samples collected from the study area^16^. After the apparent formation pressure decreased below the critical desorption pressure, the adsorbed gas began to desorb extensively and contribute to the gas production, while the cumulative gas production surpassed the value calculated using the material balance equation. The difference in the cumulative gas production increased as the formation pressure decreased. In other words, as the formation pressure decreased, the desorbed gas content of the gas produced from the core increased. The cumulative gas production was also basically linearly related with the apparent formation pressure during the low and stable production stage.

According to the material balance equation, for a closed gas reservoir, if the adsorbed gas is not considered, then the apparent formation pressure decreases linearly as the free gas production increases, as shown in the equation below^35^:

3 $$G_{free} = G_{0} \left( {1 - \frac{{Z_{i} P}}{{ZP_{i} }}} \right)$$

where $G_{0}$ is total free gas reserves in the shale formations, m^3^; $G_{free}$ is free gas amount at the reservoir pressure of $P$, m^3^; $Z_{i}$ is gas compression factor at the original reservoir pressure of $P_{i}$, dimensionless; $Z$ is gas compression factor at the reservoir pressure of $P$, dimensionless; P_i_ is initial pressure in the gas reservoir, Pa; P_i_ is reservoir pressure at time t, Pa.

Observation of the equation presented above shows that G_0_Zi/Pi and G_0_ (i.e., total free gas content) are the slope and intercept of the cumulative shale gas production–apparent pressure curve for the early stage, respectively. Therefore, G_0_ can be determined from the slope of the curve. The dotted red line in Fig. 9 signifies the calculated material balance curve for the free gas in the closed reservoir, which reflects the dynamic free gas production from the shale core and can be used as reference data to determine the free and adsorbed gas content of the gas produced at each formation pressure. The difference (approximately 1416.6 mL) between the free gas content calculated using Eq. (2) (4956 mL) and the gas content (i.e., the total free and adsorbed gas content) corresponding to the measuring point (6372 mL) is the adsorbed gas content of the gas produced from the core.

The free and adsorbed gas production patterns of the core can be further derived from the formation pressure and the patterns of the variation in the cumulative and daily gas production with the decrease in the formation pressure in conjunction with the basic theories of gas reservoir engineering^36,37^. The free gas content of the shale sample is obtained based on the pore volume and volume coefficient, while its adsorbed gas content is determined using a revised Langmuir adsorption Eq. 16 Let Pi and P denote the original formation pressure and the formation pressure at time *t*, respectively. Then, the amounts of free and adsorbed gas recovered from the core are calculated below:

4 $${\mathrm{G}}_{\mathrm{free}}=V{\phi }_{M}\frac{{T}_{\mathrm{sc}}{P}_{i}}{T{Z}_{i}{P}_{SC}}-{V\phi }_{M}\frac{{T}_{\mathrm{sc}}P}{TZ{P}_{SC}}$$

5 $${\mathrm{G}}_{\mathrm{ab}}=m*{V}_{\mathrm{L}}\frac{{p}_{i}}{{p}_{L}+{p}_{i}}\left(1-\upbeta {\rho }_{gi}\right)-m*{V}_{\mathrm{L}}\frac{p}{{p}_{L}+p}\left(1-\upbeta {\rho }_{g}\right)$$

where G_free_ is free gas production at time t, m^3^; G_ab_ is adsorbed gas production at time t, m^3^; V is shale reservoir volume, m^3^; ${\varnothing }_{M}$ is matrix porosity, dimensionless; Tsc is standard temperature, K; *P*sc is standard atmospheric pressure (0.101 MPa); T is reservoir temperature, K; $\rho_{{{\text{gi}}}}$ is gas density under initial reservoir conditions, kg/m^3^.

Therefore, the cumulative amount of gas extracted from the core is

6 $${\mathrm{G}}_{\mathrm{t}}={\mathrm{G}}_{\mathrm{free}}{+\mathrm{G}}_{\mathrm{ab}}$$

Use of the model yields a total gas content of 6703 mL for the core, of which adsorbed and free gas account for 1644 (24.5%) and 5059 mL, respectively. A comparison with the gas content results derived from the fitted curve in Fig. 9 shows the following. Of the three values, the free gas content yielded by the model is the closest to the result derived from the curve, with a difference of only 2%. The total gas content yielded by the model differs relatively markedly (by 331 mL) from the result derived from the curve, primarily due to the difference in the adsorbed gas content (228 mL; approximately 13%). This disparity can be mainly ascribed to the error in the total gas content derived from the linearly fitted curve (black portion) in Fig. 8, which occurs because as the pressure decreases, adsorbed gas desorbs at an increasing rate, resulting in a further deviation of the black line from the line denoting the linear relationship and thus a total gas content greater than 6372 mL. Therefore, the amounts of adsorbed and free gas calculated based on the porosity and isothermal adsorption curve are reliable.

**Figure 10:**
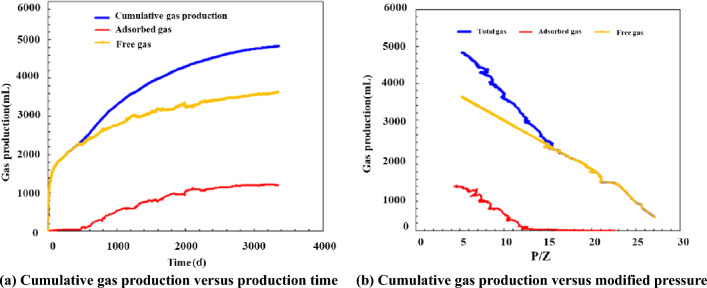
Variation in the total, adsorbed, and free gas production.

Inspection of Fig. 10 shows that the cumulative gas production curve basically overlaps the free gas production curve and is highly linearly related with the apparent mean pressure curve during the initial high-pressure stage. This phenomenon can be attributed to the nonsignificant impact of desorbed gas due to its relatively small quantity during this stage. As the pressure decreased, the adsorbed gas production increased and contributed more to the cumulative gas production, resulting in a deviation of the cumulative gas production curve from the straight line. In contrast, a high level of linearity between the free gas production and the apparent pressure persisted throughout the observation time. By the end of the production period examined in this study, the cumulative gas production had reached 4837 mL, of which free and adsorbed gas accounted for 3363 mL (71% of the total amount of free gas) and 1204 mL (73% of the total amount of adsorbed gas), respectively. The extraction of free gas was responsible for the high production during the initial stage, which was followed by the extraction of adsorbed gas. The physical simulation satisfactorily reflects the characteristics of shale gas production—high initial production, followed by a rapid decrease and stable production during the later stage. The contribution of adsorbed gas became pronounced only during the later stage, which led to a significant increase in the cumulative gas production. This phenomenon further shows that adsorbed gas constitutes an important source of shale gas that ensures a long period of stable production during the later stage of production.

## Production modeling

### Physical model

The wide range of pore sizes present in a shale reservoir leads to complex shale gas flow mechanisms that involve flow paths ranging from the molecular scale to the macroscale^38^. Shale gas production is a result of the combined action of multiple cross-scale migration mechanisms. This process can be divided into three main stages. During the first stage, the free gas in the fractures (including the secondary and artificial fractures) migrates toward the well shaft due to the decrease in the pressure in the nearby area. At the second stage, the free gas in the matrix flows toward the natural or artificial fractures due to the difference in the pressure between the matrix and fracture systems. During the third stage, the decrease in the pressure within the pores in the matrix promotes the occurrence of microflow mechanisms (e.g., the diffusion and slip of the free gas and the diffusion of the adsorbed gas on the surface), resulting in an increased gas flowability in the matrix. When the pore pressure in the matrix decreases to the critical desorption pressure for the adsorbed gas, the adsorbed gas begins to contribute to gas production by desorbing as free gas into the pores in the matrix.

**Figure 11:**
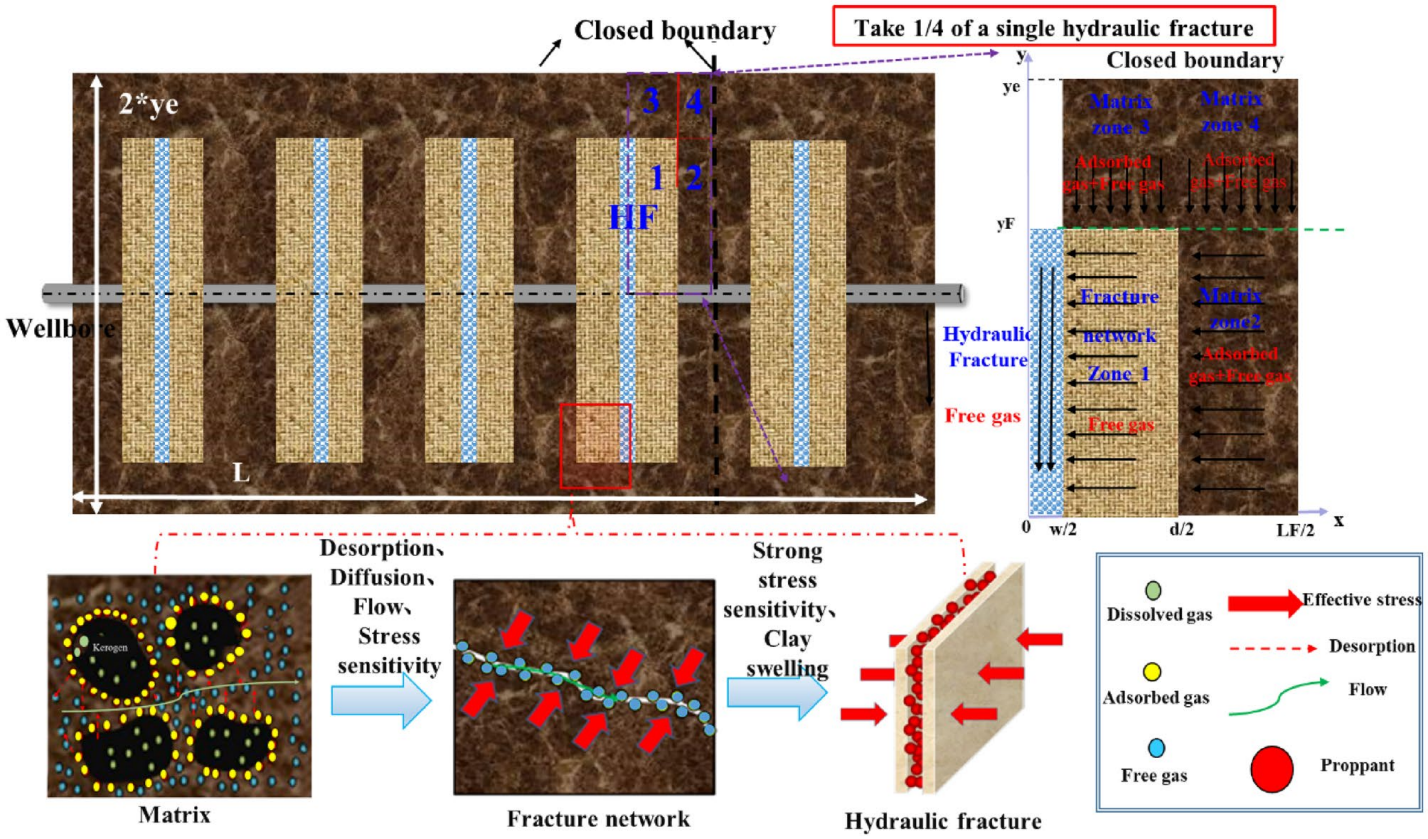
Simplified five-region physical model for a multicluster hydraulically fractured horizontal shale gas well.

Complex factors (e.g., natural fractures, brittle mineral content, and in situ stress) and various forms of hydraulic fractures induced by stimulation present challenges for accurately obtaining the characteristic parameters of a shale reservoir that describe its matrix and fracture heterogeneity. Therefore, it is assumed in this study that the forms of the main fractures in a reservoir can be characterized with a flat-plate double-wing model^39^. In addition, the fracture network and matrix region are treated as equivalent media. Some regions of the reservoir between and outside the main fractures are unfractured, resulting in a substantial interregional difference in the seepage behavior. Considering this factor, a quarter of a single main fracture is modeled. The model is divided into five regions: the main fracture in the internal region, the fracture network (region 1), the inter-main-fracture matrix (region 2), the unfractured matrix (region 3), and the matrix (region 4), as shown in Fig. 11.

The assumptions used to establish the model are detailed below:

1. Shale gas flows independently from the external regions (3 and 4) into the internal regions (1 and 2). The gas in region 2 converges toward the well shaft through region 1 and the main fracture. The migration of single-phase methane in different seepage fields is a 1D seepage process. Let *y*_*e*_ denote the half-width of the shale reservoir and well length (marked as L) represents shale reservoir length in the model. The external boundaries are closed and homogeneous with a uniform thickness.
2. The adsorbed gas in the unfractured matrix (regions 2, 3, and 4) follows the supercritical Langmuir adsorption equation. The diffusion and transition flow of the free gas and the stress sensitivity of the matrix are considered.
3. The free gas in the equivalent fracture network migrates toward the main fracture by viscous flow, and stress sensitivity is nonnegligible.
4. The main fracture in the internal region is evenly distributed and vertically symmetrical with a uniform length and completely runs through the reservoir in the vertical direction. Let *y*_F_, *w*, *L*_F_, and *d*/2 denote the half-length and width of the main fracture, the spacing between main fracture clusters, and the half-width of a single hydraulic fracture, respectively. The gas flow behavior in the main fracture in the internal region follows Darcy’s law. As the closure pressure increases, the proppant in the main fracture gradually undergoes changes (e.g., the proppant becomes elastically embedded into the wall of the main fracture and crushed), reducing the effective flow space in the main fracture.
5. The crossflow of gas from one seepage region to another is unsteady. Gas production is an isothermal seepage process. The effects of gravity and capillary forces are neglected. The bottomhole pressure in the gas well decreases from the original reservoir pressure to the predetermined output pressure during the initial stage of production, followed by constant-pressure production.
6. High-pressure physical property parameters

Because the high-pressure physical property parameters of the gas in the governing equation for seepage vary relatively considerably with temperature and pressure, the nonlinear effects cannot be neglected. A pseudopressure and a pseudotime are introduced in this study to linearize the governing equation to simplify the procedure used to solve it.

The pseudopressure is expressed as follows^38^:

7 $$\psi_{{}} = \int\limits_{0}^{P} {\frac{2P}{{\mu Z}}dP_{{}} }$$

The pseudotime is expressed as follows^38^:

8 $$t_{a} = \int\limits_{0}^{t} {\frac{{\mu_{i} C_{ti} }}{{\mu (P)C_{t} (P)}}} dt$$

where $\mu_{i}$ is gas viscosity at initial formation pressure condition, $Pa\cdot s$; $C_{ti}$ is comprehensive compression factor at initial formation pressure condition, Pa^−1^; $\mu (p)$ is gas viscosity at formation pressure of $p$,$Pa\cdot s$; $C_{ti}$ is comprehensive compression factor at formation pressure of $p$,, Pa^−1^.

2. Gas density

The density of the free-phase gas in a seepage region can be derived from the state equation for a real gas, as shown below:

9 $$\rho_{g} = \frac{{P_{{}} M}}{ZRT}$$

where M is molar mass of methane (16 g/mol); R is universal gas constant (8.314 J/mol/K); *T*—reservoir temperature, K.

3. Supercritical adsorption–desorption model for gas

Analysis of the isothermal adsorption and development simulation test results presented earlier reveals the following phenomena that occur during the extraction of gas from a shale gas reservoir through depressurization. When the formation pressure is higher than the critical desorption pressure, adsorbed gas remains basically unextracted, while the gas well primarily produces free gas. When the formation pressure is lower than the critical desorption pressure, the gas originally in the adsorbed state desorbs and complements the free gas extracted from the reservoir, resulting in a decreased rate at which the pressure decreases and therefore ensuring a long period of stable production from the shale gas well during the later stage. There is a point on the high-pressure isothermal adsorption curve that corresponds to the maximum adsorption. When the pressure is higher than the critical pressure at this point, the adsorption is negatively correlated with the pressure^40,41^. The conventional Langmuir adsorption equation is unsuitable for describing high-pressure isothermal adsorption curves for shale reservoirs (Fig. 1). In this section, the following excess high-pressure isothermal adsorption model established based on the adsorbed-phase volume theory^40^ is used:

10 $$q_{ad} = \frac{{P_{sc} M}}{{Z_{sc} RT_{sc} }}V_{L} \frac{{P_{i} }}{{P_{i} + P_{L} }}\left(1 - \frac{{\rho_{g} }}{{\rho_{a} }}\right)$$

where *q*_*ad*_ is supercritical excess amount of adsorbed shale gas per unit volume of the matrix, kg/m^3^; *Z*_*sc*_ is compressibility factor for an ideal gas, dimensionless.

4. Apparent permeability model

During the later stage of production of a shale gas reservoir, the flow regime in the micro- and nanopores in the matrix is not controlled by a single migration mechanism but instead is a result of the coupling of multiple flow mechanisms (e.g., viscous flow, diffusion, and transition flow). Establishing a multiscale, multiflow-regime apparent permeability model to reveal micro- and nanoscale gas flow patterns is a key scientific problem associated with production modeling. In this study, the stress sensitivity of the matrix and the desorption of gas in the matrix are coupled. Then, the apparent permeability model established by Wu et al.^42^ for micro- and nanopores in shale matrices based on molecular dynamics theories is used to superpose the viscous flow and Knudsen diffusion of real gas molecules with weighting coefficients:

11 $$K_{ma} = \left( {\frac{1}{{K_{ne} + 1}}K_{m0} + \frac{{K_{ne} }}{{K_{ne} + 1}}C_{g} D\mu } \right) \cdot \left( {1 + \frac{{C_{d} }}{{C_{d} + C_{g} + C_{m} }}} \right)$$

The effective Knudsen number is given below:

12 $$K_{ne} = \frac{\lambda }{{r_{e} }} = \frac{{K_{B} T}}{{r_{e} \sqrt 2 \pi d^{2} \overline{p} }}$$

The effective hydraulic flow radius that accounts for stress sensitivity and desorption is expressed as follows:

13 $$r_{e} = r_{0} e^{{ - \gamma_{m} (p_{i} - p_{m} )/2}} - \theta d_{{CH_{4} }}$$

The coverage of gas adsorbed onto the wall surface of the pores in the matrix is calculated below:

14 $$\theta = \frac{{p_{m} }}{{p_{m} + p_{L} }}(1 - \frac{{\rho_{g} }}{{\rho_{a} }})$$

where $K_{ma}$ is effective apparent matrix permeability, m^2^; $K_{ne}$ is effective Knudsen number, dimensionless;$K_{m0}$ is matrix intrinsic permeability, m^2^; $C_{g}$ is gas compressibility coefficient, Pa^−1^; $D$ is gas diffusion coefficient, m^2^/s; $C_{d}$ is compressibility factor for desorbed gas in the matrix, Pa^–1^; $C_{m}$ is matrix compressibility coefficient, Pa^−1^; $D$ is gas diffusion coefficient, m^2^/s; $C_{d}$ is compressibility factor for desorbed gas in the matrix, Pa^–1^; $\lambda$ is length of the free path of methane molecules at any arbitrary temperature and pressure, m; $r_{e}$ is effective hydraulic flow radius, m; $r_{0}$ is initial hydraulic flow radius, m; $p_{m}$ is shale matrix pressure, Pa; $\gamma_{m}$ is shale matrix stress sensitivity, Pa^−1^; $\overline{p}$ is average pressure in the reservoir, Pa; $K_{B}$ is Boltzmann constant (1.38065 × 10^–23^ J/K); $d$ is collision diameter of methane molecules, m; $d_{{CH_{4} }}$ is diameter of methane molecules, m.

5. Pressure sensitivity effect

When gas is extracted from a fracture network, the non-proppant-supported secondary fractures may be closed due to stress sensitivity, resulting in a significantly lower permeability of the secondary fracture network. Therefore, an empirical stress sensitivity model in an exponential form^41^, given below, is used in this study to characterize the effects of the stress sensitivity of secondary fractures on gas flowability.

15 $$K_{f} = K_{fi} e^{{ - \gamma (\psi_{e} - \psi_{f} )}}$$

where $K_{f}$ is permeability that accounts for pressure sensitivity, m^2^; $K_{fi}$ is permeability at the initial time, m^2^; γ—pseudo permeability modulus, Pa^2^/(Pa^2^·s).

Based on the dimensionless parameters defined in Table 2, the system of governing equations for seepage is converted to dimensionless seepage equations to solve the dimensionless productivity model.

**Table 2:** Definitions of dimensionless parameters.

Dimensionless parameter	Equation of definition	Dimensionless parameter	Equation of definition
Dimensionless pseudopressure	$\psi_{D} = \frac{{\psi_{i} - \psi }}{{\psi_{i} - \psi_{wf} }}$	Dimensionless production	$\frac{1}{{q_{D} }} = \frac{{T_{sc} K_{F} \sqrt {A_{cw} } (\psi_{i} - \psi_{wf} )}}{{p_{sc} q_{sc} T}}$
Dimensionless pseudotime	$t_{aD} = \frac{{K_{F} t_{a} }}{{\mu (\varphi_{1} C_{t1} + \varphi_{2} C_{t2} + \varphi_{3} C_{t3} )A_{cw} }}$	Storativity ratio	$w_{i} = \frac{{\varphi_{i} C_{ti} }}{{\varphi_{1} C_{t1} + \varphi_{2} C_{t2} + \varphi_{3} C_{t3} }}(i = 1,2,3)$
Dimensionless length in the *x*-direction	$x_{D} = \frac{2x}{{L_{f} }}$	Coefficient of mass transfer from region 4 to region 2	$\lambda_{24} = \frac{{12K_{4} }}{{L_{F}^{2} K_{2} }}A_{cw}$
Dimensionless length in the *y*-direction	$y_{D} = \frac{y}{{\sqrt {A_{cw} } }}$	Coefficient of mass transfer from region 3 to region 1	$\lambda_{13} = \frac{{12K_{1} }}{{L_{F}^{2} K_{3} }}A_{cw}$
Conductivity coefficient for region 3	$\eta_{3D} = \frac{{K_{F} }}{{\varphi_{1} C_{t1} + \varphi_{2} C_{t2} + \varphi_{F} C_{tF} }}\frac{{\varphi_{3} C_{t3} }}{{K_{3a} }}$	Coefficient of mass transfer from region 1 to region F	$\lambda_{1F} = \frac{{12K_{1} }}{{L_{F}^{2} K_{F} }}A_{cw}$
Conductivity coefficient for region 4	$\eta_{4D} = \frac{{K_{F} }}{{\varphi_{1} C_{t1} + \varphi_{2} C_{t2} + \varphi_{F} C_{tF} }}\frac{{\varphi_{4} C_{t4} }}{{K_{4a} }}$	Dimensionless flow conductivity of the reservoir	$R_{CD} = \frac{{K_{1} d}}{{K_{2} L_{F} }}$

Where $\psi_{i}$ is initial formation pseudo-pressure, Pa^2^/(Pa$\cdot$s); $\psi$ is the formation pseudo-pressure, Pa^2^/(Pa$\cdot$s); $K_{F}$ is hydraulic fracture permeability, m^2^; $\varphi_{2}$ is porosity of the matrix zone 2, dimensionless; $\varphi_{1}$ is porosity of fracture network, dimensionless; $\varphi_{3}$ is porosity of matrix zone 3. m^2^; $C_{t3}$ is comprehensive compressibility of matrix zone 3, Pa^−1^; $C_{t2}$ is comprehensive compressibility of matrix zone 2, Pa^−1^; $C_{t1}$ is comprehensive compressibility of fracture network, Pa^−1^; $t_{a}$ is pseudo-time, s; $L_{f}$ is hydraulic fracture spacing, m; $A_{cw}$ is wellbore crossflow area, m^2^; $\varphi_{F}$ is porosity of hydraulic fracture, m; $C_{tF}$ is comprehensive compressibility of hydraulic fracture, Pa^−1^; $K_{3a}$ is apparent permeability of matrix zone 3, m^2^; $K_{1}$ is fracture network permeability, m^2^; $K_{2}$ is matrix zone 2 permeability, m^2^;$d$ is the width of stimulated reservoir zone, m.

### Mathematical model

1. Governing equation for seepage in the matrix in region 4

The gas in the matrix in region 4 converges along the *y*-direction into the matrix in region 2 by unsteady crossflow. Considering the supercritical desorption of the adsorbed gas and the Knudsen diffusion and viscous flow of the gas in the nanopores in the shale matrix, a closed external boundary and a continuous pressure at the internal boundary are used^43^.

Based on the law of conservation of mass, a governing equation for seepage in the matrix is obtained, as shown below:

16 $$\frac{{\partial (\rho_{g} \varphi_{{4}} )}}{\partial t} + \frac{{\partial (1 - \varphi_{{4}} )q_{ad} }}{\partial t} = - \frac{\partial }{\partial y}(\rho_{g} v_{{4}} )$$

The seepage velocity of the gas in the matrix can be derived, as shown below:

17 $$v_{{4}} = - \frac{{K_{{{4}a}} }}{{\mu_{{4}} }}\frac{{\partial P_{{4}} }}{\partial y}$$

Use of Eq. (17) to transform the left-hand side of Eq. (16) yields

18 $$\frac{{\partial \left( {\rho_{{\text{g}}} \varphi_{{4}}^{{}} } \right)}}{\partial t} + \frac{{\partial (1 - \varphi_{{4}}^{{}} )q_{ad} }}{\partial t} = \rho_{{\text{g}}} \varphi_{{4}}^{{}} \left[ {(\frac{1}{{\varphi_{{4}}^{{}} }}\frac{{\partial \varphi_{{4}}^{{}} }}{{\partial P_{{4}} }} + \frac{1}{{\rho_{g} }}\frac{{\partial \rho_{g} }}{{\partial P_{{4}} }})\frac{{\partial P_{4} }}{\partial t}{ + (}V_{L} \frac{{(1 - \varphi_{4} )TZP_{sc} }}{{\varphi_{4} Z_{sc} T_{sc} }}\frac{{P_{L} }}{{P_{4} (P_{4} + P_{L} )^{2} }}(1 - \frac{{\rho_{{\text{g}}} }}{{\rho_{a} }}))} \right]\frac{{\partial P_{4} }}{\partial t}$$

The above equation does not consider the effects of formation compression. Compressibility factors for gas in the free and supercritical desorbed states are defined below^43^:

19 $$C_{{g{4}}} = \frac{1}{{\rho_{g} }}\frac{{\partial \rho_{g} }}{{\partial P_{{4}} }}$$

20 $$C_{{d{4}}} = V_{L} \frac{{(1 - \varphi_{{4}} )TZP_{sc} }}{{\varphi_{{4}} Z_{sc} T_{sc} }}\frac{{P_{L} }}{{P_{{4}} (P_{{4}} + P_{L} )^{2} }}(1 - \frac{{\rho_{{\text{g}}} }}{{\rho_{a} }})$$

Adding the excess gas adsorption in the form of the compressibility factor for gas in the desorbed state into the compressibility factor for gas in the free state yields a comprehensive compressibility factor for gas in the matrix in the external region, *C*_t4_:

21 $$C_{{t{4}}} = C_{{g{4}}} + C_{{d{4}}}$$

Combining Eqs. (19), (20), and (21) and substituting the result into Eq. (18) transforms it to

22 $$\frac{{\partial \left( {\rho_{{\text{g}}} \varphi_{{4}}^{{}} } \right)}}{\partial t} + \frac{{\partial (1 - \varphi_{{4}}^{{}} )q_{ad} }}{\partial t} = \rho_{{\text{g}}} \varphi_{{4}}^{{}} C_{{t{4}}} \frac{{\partial P_{{4}} }}{\partial t}$$

Substitution of Eqs. (17) and (22) into Eq. (16) gives

23 $$\rho_{{\text{g}}} \varphi_{{4}}^{{}} C_{{t{4}}} \frac{{\partial P_{{4}} }}{\partial t}{ = }\frac{\partial }{\partial y}(\rho_{{\text{g}}} \frac{{K_{{{4}a}} }}{{\mu_{{4}} }}\frac{{\partial P_{{4}} }}{\partial y})$$

where $\varphi_{{4}}^{{}}$ is porosity of matrix zone 4, dimensionless; $C_{{t{4}}}$ is the matrix comprehensive compressibility coefficient, Pa^−1^; $P_{{4}}$ is pore pressure in matrix zone 4, Pa; $t$ is production duration, s; $K_{{{4}a}}$ is apparent permeability of matrix zone 4, m^2^; $\mu_{{4}}$ is gas viscosity, $Pa\cdot s$; $C_{{{\text{g4}}}}$ is gas compressibility, Pa^−1^; $C_{{{\text{d4}}}}$ is modified supercritical desorption gas compression coefficient of matrix zone 4, Pa^−1^; $C_{{{\text{f4}}}}$ is formation compressibility, Pa^−1^; $\varphi_{{4}}$ is porosity of the matrix zone 4, dimensionless; $K_{4a}$ is apparent permeability of matrix zone 4, m^2^; $K_{4i}$ is intrinsic permeability of matrix zone 4, m^2^.

For simplicity, Eq. (23) is transformed into a pseudo-function with dimensionless variables, which is subsequently simplified to yield Eq. (24):

24 $$\frac{{\partial \psi_{4D} }}{{\partial t_{{\text{a}}} }} = \frac{1}{{\eta_{4D} }}\frac{{\partial^{2} \psi_{4D} }}{{\partial y^{2} }}$$

The pressure in the seepage field in the external region at the initial time is equivalent to the initial formation pressure, as shown below:

25 $$P_{{4}} (t = 0) = P_{e}$$

26 $${\text{External boundary}}:\,\left. {\frac{{\partial P_{{4}} }}{\partial y}} \right|_{{y = y_{e} }} = 0$$

27 $${\text{Internal boundary}}:\,P_{{4}} (y = y_{F} ) = P_{{2}} (y = y_{F} )$$

Equations (25)–(27) are transformed into the following forms of dimensionless boundary conditions:

28 $$\psi_{4D} (y_{D} ,0) = 0$$

29 $$\psi_{4D} (y_{FD} ,t_{aD} ) = \psi_{2D}$$

30 $$\left. {\frac{{\partial \psi_{4D} (y_{D} ,t_{{{\text{a}}D}} )}}{{\partial y_{D} }}} \right|_{{y_{D} = y_{{{\text{eD}}}} }} = 0$$

By performing a Laplace transform on the dimensionless pseudopressure in Eq. (24), the following form of pseudopressure in the Laplace space is obtained:

31 $$\zeta_{LD} = L[\zeta_{D} ] = \int_{0}^{\infty } {\zeta_{D} e^{{ - st_{D} }} } dt_{D}$$

Multiplying both sides of Eq. (31) by ${e}^{-stD}$ and subsequently integrating the resulting equation over the dimensionless time *t*_a*D*_ from zero to infinity gives

32 $$\int_{0}^{\infty } {e^{{ - st_{{{\text{a}}D}} }} } \frac{{\partial \zeta_{{{4}D}} }}{{\partial t_{{{\text{a}}D}} }}dt_{{{\text{a}}D}} = \frac{1}{{\eta_{{{4}D}} }}\int_{0}^{\infty } {e^{{ - st_{{{\text{a}}D}} }} } \frac{{\partial^{2} \zeta_{{{4}D}} }}{{\partial y_{D}^{2} }}dt_{{{\text{a}}D}}$$

The integral term on the left-hand side of Eq. (32) is simplified to

33 $$\int_{0}^{\infty } {e^{{ - st_{{{\text{a}}D}} }} } \frac{{\partial \zeta_{{{4}D}} }}{{\partial t_{{{\text{a}}D}} }}dt_{{{\text{a}}D}} = \int_{0}^{\infty } {e^{{ - st_{{{\text{a}}D}} }} } d\zeta_{{{4}D}} = e^{{ - st_{{{\text{a}}D}} }} \zeta_{{{4}D}} \left| {_{0}^{\infty } } \right. + s\int_{0}^{\infty } {e^{{ - st_{{{\text{a}}D}} }} } \zeta_{{{4}D}} dt_{{{\text{a}}D}} = s\zeta_{{L{4}D}}$$

The integral term on the right-hand side of Eq. (32) is simplified to

34 $$\int_{0}^{\infty } {e^{{ - st_{{{\text{a}}D}} }} } \frac{{\partial^{2} \zeta_{{{4}D}} }}{{\partial y_{D}^{2} }}dt_{{{\text{a}}D}} = \frac{{\partial^{2} \int_{0}^{\infty } {e^{{ - st_{{{\text{a}}D}} }} \zeta_{{{4}D}} dt_{{{\text{a}}D}} } }}{{\partial y_{D}^{2} }} = \frac{{\partial^{2} \zeta_{{L{4}D}} (y_{D} ,s)}}{{\partial y_{D}^{2} }}$$

Substituting Eqs. (33) and (34) into Eq. (32) yields the following dimensionless governing equation for seepage in the matrix in region 4 in the Laplace form:

35 $$\frac{{\partial^{2} \zeta_{{L{4}D}} }}{{\partial y_{D}^{2} }} = s\eta_{{{4}D}} \zeta_{{L{4}D}}$$

The boundary conditions in the Laplace form are simplified to

36 $$\zeta_{{L{4}D}} (y_{FD} ,s) = \zeta_{{L{2}D}}$$

37 $$\left. {\frac{{\partial \zeta_{{L{4}D}} (y_{D} ,s)}}{{\partial y_{D} }}} \right|_{{y_{D} = y_{{{\text{eD}}}} }} = 0$$

Solving Eqs. (35)–(37) simultaneously yields a pseudo-pressure solution for the dimensionless seepage model for the matrix in region 4 in the Laplace space:

38 $$\zeta_{{L{4}D}} (y_{D} ,s) = \zeta_{{L{2}D}} \frac{{\cosh \left[ {\sqrt {s\eta_{{{4}D}} } (y_{{{\text{e}}D}} - y_{D} )} \right]}}{{\cosh \left[ {\sqrt {s\eta_{{{4}D}} } (y_{{{\text{e}}D}} - y_{FD} )} \right]}}$$

2. Governing equation for seepage in the matrix in region 3

Similar to the gas seepage pattern in the matrix in region 4, gas in the matrix in region 3 converges along the *y*-direction toward the matrix in region 1 by Knudsen diffusion, viscous flow, and unsteady crossflow. A dimensionless governing equation for seepage in the matrix in region 3 can be derived from the mass conservation, motion, and state equations, as shown below:

39 $$\frac{{\partial \psi_{3D} }}{{\partial t_{{{\text{a}}D}} }} = \frac{1}{{\eta_{3D} }}\frac{{\partial^{2} \psi_{3D} }}{{\partial y_{D}^{2} }}$$

Because the external boundary is closed and a continuous pressure is present at the internal boundary, the dimensionless initial and boundary conditions expressed in Eqs. (40)–(42) hold:

40 $$\psi_{3D} (y_{D} ,0) = 0$$

41 $$\psi_{3D} (y_{FD} ,t_{aD} ) = \psi_{1D}$$

42 $$\left. {\frac{{\partial \psi_{3D} (y_{D} ,t_{aD} )}}{{\partial y_{D} }}} \right|_{{y_{D} = y_{{{\text{eD}}}} }} = 0$$

A dimensionless pseudopressure solution in the Laplace form for the matrix in region 3, shown in Eq. (43), is obtained through a Laplace transform using a procedure similar to that used to solve the dimensionless governing equation for flow in the matrix in region 4.

43 $$\zeta_{{L{3}D}} (y_{D} ,s) = \zeta_{{L{1}D}} \frac{{\cosh \left[ {\sqrt {s\eta_{{{3}D}} } (y_{eD} - y_{D} )} \right]}}{{\cosh \left[ {\sqrt {s\eta_{{{3}D}} } (y_{eD} - y_{FD} )} \right]}}$$

3. Governing equation for seepage in the matrix in region 2

Gas in the matrix in region 2 flows into the matrix in region 1 along the *x*-direction by diffusion and viscous flow. In addition, the external boundary is closed, while a continuous pressure is present at the internal boundary in regions 2 and 1. Considering these factors, a dimensionless governing equation for seepage in the matrix in region 2 is established, as shown below:

44 $$\frac{{\partial^{2} \psi_{2D} }}{{\partial x_{D}^{2} }} = \frac{{3w_{2} }}{{\lambda_{12} }}\frac{{\partial \psi_{2D} }}{{\partial t_{{{\text{a}}D}} }} - \left. {\frac{6}{{\lambda_{24} y_{FD} }}\frac{{\partial \psi_{4D} }}{{\partial y_{D} }}} \right|_{{y = y_{FD} }}$$

The external boundary allows no flow. The pseudopressure in the fracture network at the internal boundary is identical to that in the main fracture. The pseudopressure in the seepage field in the fracture network at the initial time is equivalent to the initial formation pseudopressure. Thus, the initial boundary conditions can be expressed as follows:

45 $${\text{Initial condition}}:\,\psi_{2D} (x_{D} ,0) = 0$$

46 $${\text{External boundary}}:\,\left. {\frac{{\partial \psi_{2D} (x_{D} ,t_{aD} )}}{{\partial x_{D} }}} \right|_{{x_{D} = 1}} = 0$$

47 $${\text{Internal boundary}}\psi_{2D} (\frac{{d_{D} }}{2},t_{{{\text{a}}D}} ) = \psi_{1D}$$

A Laplace transform is performed to derive a dimensionless pseudopressure solution for the governing equation for flow in the matrix in region 2:

48 $$\zeta_{{{\text{L2}}D}} ({\text{x}}_{D} ,s) = \zeta_{L1D} \frac{{\cosh (\sqrt {\alpha_{2} } (1 - x_{D} ))}}{{\cosh (\sqrt {\alpha_{2} } (1 - \frac{{d_{D} }}{2}))}}$$

where

49 $$\alpha_{{2}} = \frac{{{3}w_{{2}} s}}{{\lambda_{{{12}}} }}{ + }\frac{{{6}\sqrt {s\eta_{4D} } }}{{\lambda_{24} y_{FD} }}\tanh (\sqrt {s\eta_{4D} } (y_{eD} - y_{FD} ))$$

4. Governing equation for seepage in the fracture network in region 1

Gas in the fracture network in region 1 converges along the *x*-direction toward the well shaft by viscous flow. The flow at the external boundary and in region 2 is continuous, while the pressure at the internal boundary and in the main fracture is continuous. Considering the stress sensitivity of the fracture network region, a dimensionless governing equation for seepage in the fracture network in region 1 can be derived from the mass conservation, motion, and state equations:

50 $$\frac{{\partial^{2} \psi_{1D} }}{{\partial x_{D}^{2} }} = \frac{{3w_{1} }}{{\lambda_{1F} }}\frac{{\partial \psi_{1D} }}{{\partial t_{aD} }} - \left. {\frac{6}{{\lambda_{13} y_{FD} }}\frac{{\partial \psi_{3D} }}{{\partial y_{D} }}} \right|_{{y = y_{FD} }}$$

Equations (51)–(53) show the boundary conditions:

51 $$\text{Initial condition: } \psi_{1D} (x_{D} ,0) = 0$$

52 $${\text{External boundary condition}}:\psi_{1D} (0,t_{aD} ) = \psi_{FD}$$

53 $${\text{Internal boundary condition}}:\left. {\frac{{\partial \psi_{1D} (x_{D} ,t_{aD} )}}{{\partial x_{D} }}} \right|_{{x_{D} = \frac{{d_{D} }}{2}}} = \frac{{\lambda_{12} }}{{\lambda_{1F} }}\left. {\frac{{\partial \psi_{2D} (x_{D} ,t_{aD} )}}{{\partial x_{D} }}} \right|_{{x_{D} = \frac{{d_{D} }}{2}}}$$

Similarly, a Laplace transform is performed to produce a pseudo-pressure solution for the dimensionless governing equation for seepage in the fracture network region:

54 $$\zeta_{{{\text{L1}}D}} (x_{D} ,s) = \zeta_{LFD} \frac{{c3\sinh (\sqrt {\alpha_{1} } (x_{D} - \frac{{d_{D} }}{{2}})) + \cosh (\sqrt {\alpha_{1} } (x_{D} - \frac{{d_{D} }}{{2}}))}}{{ - c3\sinh (\sqrt {\alpha_{1} } \frac{{d_{D} }}{{2}}) + \cosh (\sqrt {\alpha_{1} } \frac{{d_{D} }}{{2}})}}$$

where

55 $$c3 = \frac{{\lambda_{{{12}}} }}{{\lambda_{{{\text{1F}}}} }}\sqrt {\frac{{\alpha_{2} }}{{\alpha_{1} }}} \tanh (\sqrt {\alpha_{2} } (\frac{{d_{D} }}{{2}} - 1))$$

56 $$\alpha_{{1}} = \frac{{{3}w_{{1}} s}}{{\lambda_{{{\text{1F}}}} }}{ + }\frac{{{6}\sqrt {s\eta_{3D} } }}{{\lambda_{13} y_{FD} }}\tanh (\sqrt {s\eta_{3D} } (y_{eD} - y_{FD} ))$$

5. Governing equation for seepage in the main fracture in the internal region

Gas in the main fracture migrates toward the well shaft by viscous flow. A dimensionless governing equation for seepage in the main fracture is established based on the law of conservation of mass in conjunction with the motion equation, as shown below:

57 $$\frac{{\partial^{2} \psi_{FD} }}{{\partial {\text{y}}_{D}^{2} }} = w_{F} \frac{{\partial \psi_{FD} }}{{\partial t_{{{\text{a}}D}} }} - \left. {\frac{{\lambda_{1F} e^{{\gamma_{1D} \psi_{{{1}D}} }} }}{3}\frac{{\partial \psi_{1D} }}{{\partial x_{D} }}} \right|_{{x_{D} = \frac{{d_{D} }}{{2}}}}$$

Considering that the gas well produces gas at a constant bottomhole pressure and that the external boundary is closed, the following dimensionless initial boundary conditions hold:

58 $${\text{Initial condition}}:\,\psi_{FD} (y_{D} ,0) = 0$$

59 $${\text{External boundary}}:\left. {\frac{{\partial \psi_{FD} (y_{D} ,t_{aD} )}}{{\partial t_{aD} }}} \right|_{{y_{D} = y_{FD} }} = 0$$

60 $${\text{Internal}}\,{\text{boundary}}\,\psi_{FD} (0,t_{{{\text{a}}D}} ) = 1$$

A pseudopressure solution in the Laplace space for the derived dimensionless governing equation for seepage in the main fracture is given below:

61 $$\zeta_{{{\text{LF}}D}} (y_{D} ,s) = \frac{{\cosh (\sqrt {\alpha_{4} } (y_{D} - y_{FD} ))}}{{\cosh (\sqrt {\alpha_{4} } y_{FD} )}}$$

where

62 $$\alpha_{4} {\text{ = w}}_{F} s - \frac{{\lambda_{1F} \alpha_{3} }}{{3\gamma_{fD} }}(1 - e^{{ - \gamma_{fD} }} )$$

63 $$\alpha_{3} = \frac{{\sqrt {\alpha_{1} } c3\cosh (\sqrt {\alpha_{1} } (x_{D} - \frac{{d_{D} }}{{2}})) + \sqrt {\alpha_{1} } \sinh (\sqrt {\alpha_{1} } (x_{D} - \frac{{d_{D} }}{{2}}))}}{{ - c3\sinh (\sqrt {\alpha_{1} } \frac{{d_{D} }}{{2}}) + \cosh (\sqrt {\alpha_{1} } \frac{{d_{D} }}{{2}})}}$$

Let *N* denote the number of hydraulic fractures in the shale gas well. Then, the dimensionless gas production rate at the bottom of the shale gas well can be expressed in the Laplace space as follows:

64 $${\text{q}}_{LD} = - \frac{N}{2\pi }\frac{{\partial \zeta_{LFD} }}{{\partial y_{D} }}\left| {_{{y_{D} = 0}}^{{}} } \right. = \frac{{N\sqrt {\alpha_{4} } }}{2\pi s}\tanh (y_{FD} \times \sqrt {\alpha_{4} } )$$

Subsequently, a real-space semianalytical dimensionless gas production rate is obtained through a Stehfest numerical inversion^40^. Finally, Newton’s iteration method can be used to determine the real-space gas production from the gas well at a constant pressure.

## Model validation and prediction

### Fitting physical simulation test results

To examine the effectiveness of the model, the matrix part of the model is validated based on the physical simulation test results. The developed mathematical model is first simplified by assuming that the hydraulic fracture and fracture network regions have infinite flow conductivity and by neglecting the gas flow in the reservoir in the *y*-direction in the model. Then, a 1D gas production pattern is simulated for the matrix at the core scale. Subsequently, the gas production test data for the matrix core are fitted. Finally, the model-fitted parameter (Table 3) is inverted to validate the model. (* indicates a model-fitted parameter).

**Table 3:** Test parameters and model-fitted parameter.

Parameter	Value	Parameter	Value
Initial pressure/MPa	30	Matrix porosity	0.023
Test temperature/K	300	Matrix permeability/mD	0.00005
Core length/m	0.16	Langmuir volume/(m^3^/t)*	2.1
Core seepage area/m^2^	0.78	Langmuir pressure/MPa	10

**Figure 12:**
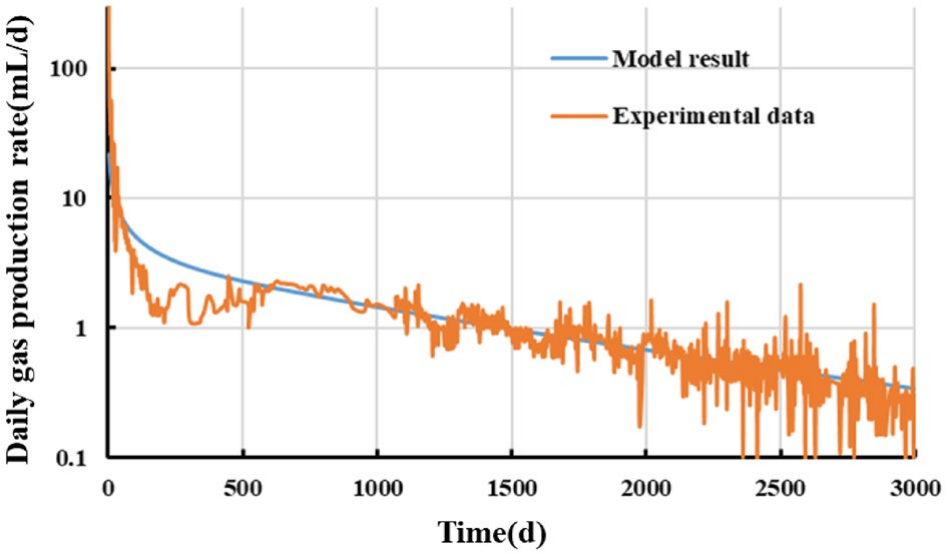
Comparison of measured and model-fitted daily gas production data.

**Figure 13:**
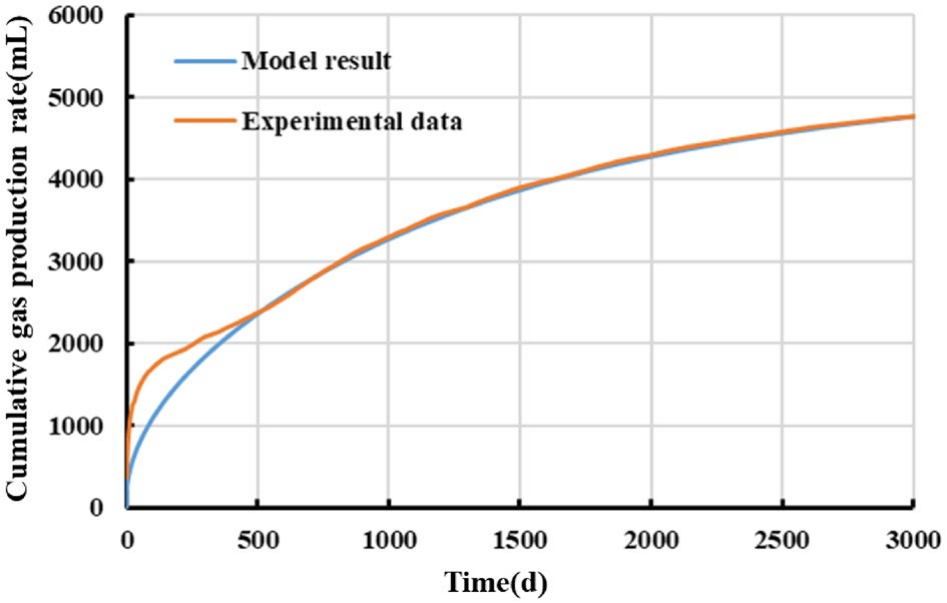
Comparison of measured and model-fitted cumulative gas production data.

Figures 12 and 13 compare the measured and model-fitted gas production curves. Overall, a relatively high goodness-of-fit can be observed between the gas production curves yielded by the developed five-region productivity model and the test. The model yields a cumulative gas production of 4756 mL, which differs by less than 5% from the actual value for the gas well after 3000 days of production. This finding validates the soundness and feasibility of the coupling of the high-pressure adsorption, apparent permeability, and stress sensitivity models in the developed model, while showing that the model can be used to theoretically analyze the productivity of shale gas wells and predict their production patterns. Note that there is a marked difference between the actual and model-fitted production curves for a period of time during the initial stage, which can be primarily ascribed to the overly fast decrease in the outlet pressure during production. A relatively high goodness-of-fit can be observed between the measured and model-fitted curves for the stable period of production during the later stage.

### Single-well simulation and production prediction

The productivity model developed and validated based on the physical simulation test results earlier was used to fit the dynamic production data for a multistage-fractured horizontal well (NH3-5–1) in the Changning Shale Gas Demonstration Area in the Sichuan Basin and to analyze its full-life-cycle dynamic production. Table 4 summarizes the relevant geological parameters and well completion parameters. (* indicates a model-fitted parameter).

**Table 4:** Geological parmeters of the gas reservoir and parameters of the horizontal well.

Parameter	Value	Parameter	Value
Initial reservoir pressure/MPa	70	Number of fractured sections	30
Reservoir temperature/K	390	Hydraulic fracture half-length/m*	100
Reservoir thickness/m	16	Hydraulic fracture width/m*	0.003
Matrix porosity	0.043	Interwell spacing/m	300
Matrix permeability/mD	0.000165	Length of the horizontal section of the gas well/m	1465
Fracture network porosity	0.055	Bottomhole flowing pressure/MPa	3
Fracture network permeability/mD*	1.251	Rock density/(kg/m^3^)	2600
Hydraulic fracture permeability/mD*	50	Stress sensitivity coefficient/MPa^–1^	0.14
Langmuir volume/(m^3^/t)	2.5	Langmuir pressure/MPa	12
Half-width of the hydraulic fracture region/m*	3	Number of fracture clusters	3

*Indicates a model-fitted parameter.

**Figure 14:**
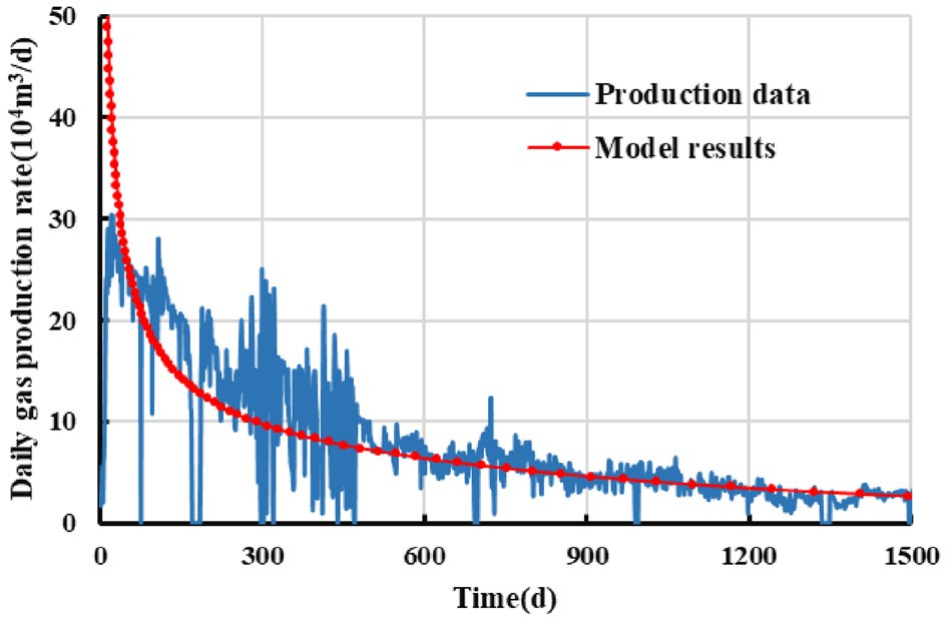
Fit for the daily gas production of well NH3-5-1.

**Figure 15:**
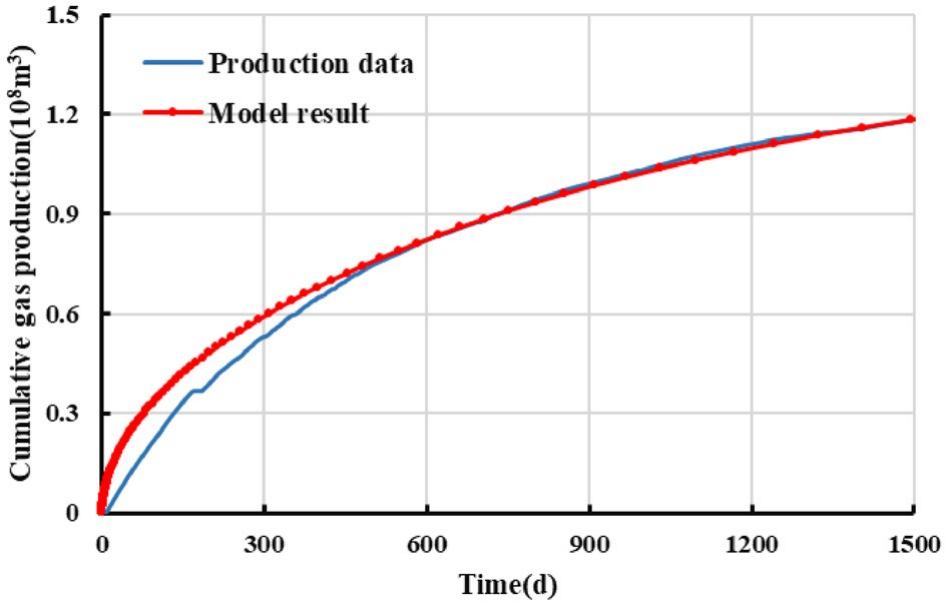
Fit for the cumulative gas production of well NH3-5-1.

Observation of the model-fitted gas production curves in Figs. 14 and 15 reveals the following. The production of the gas well lasted for 1500 days. The daily production fluctuated relatively appreciably but, overall, decreased in a relatively markedly monotonic pattern. Over the first 900 days, the daily gas production rate decreased from 300,000 to approximately 50,000 m^3^, while the cumulative gas production reached 0.98 × 10^8^ m^3^. The gas well had basically entered the stable production stage during this time period. Generally similar trends can be observed between the gas production curves yielded by the developed productivity model and the actual dynamic production curves. At first months of production, production data is lower than the model result, which can be possibly attributable to the fracturing fluid back flow, excessive choking and/or lack of takeaway capacity ^[^^44^^]^. The model yields a cumulative gas production of 0.98 × 10^8^ m^3^, which differs by approximately 1.27% from the actual value after 1500 days of production. These findings show that the developed productivity model is reliable and can be used to accurately generate productivity and dynamic production pattern estimates for gas wells.

**Figure 16:**
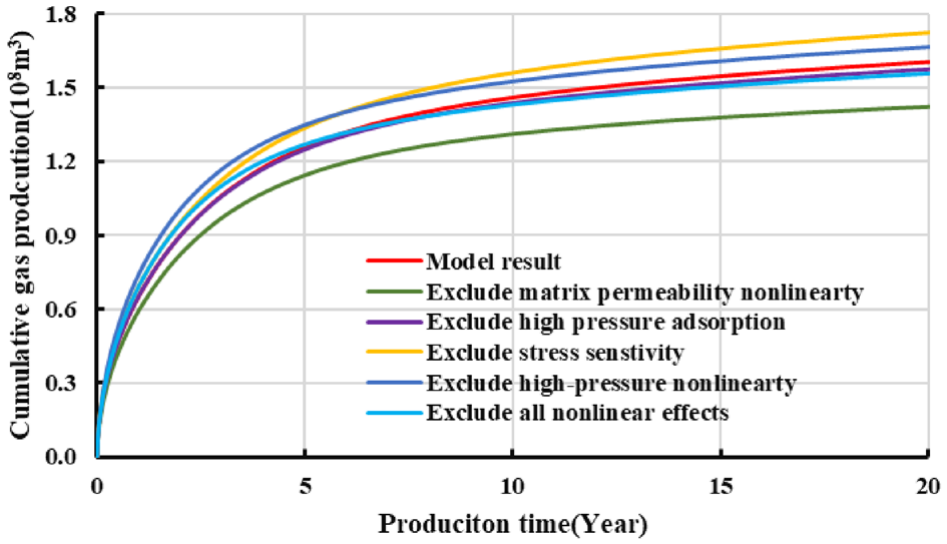
Diagram of the daily gas production and time for different models.

**Figure 17:**
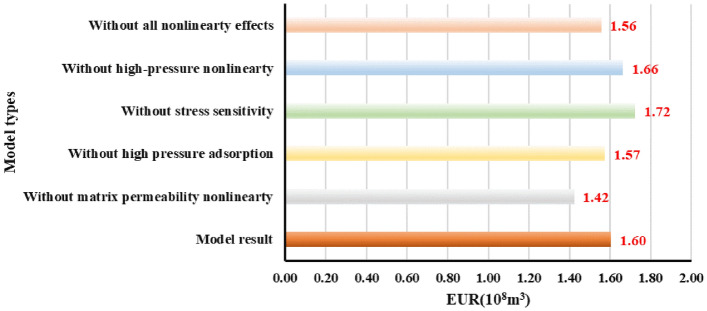
Diagram of the EUR comparison for different models.

Figures 16 and 17 show that when the non-linear gas flow mechanism is not considered in the production model, the predicted 20-year-production predicted by the model can be underestimated by 11.3%, indicating diffusion and desorption is significant to the later-stage-productivity; the calculated EUR of considering stress sensitivity is 7.5% lower than that model predictions without involving stress sensitivity. Likewise, the model considering the high-pressure adsorption predicts that EUR is 1.87% higher than that of only considering Langmuir adsorption. If the above comprehensive factors are considered in the model, the EUR is about 2.5% higher than that of the linear production model. To sum up, the comprehensive factors considered in the production model is conducive to enhance the accuracy of predicting the medium and long-term productivity. It aims to provide reliable reference to accurately estimate shale gas well production at different production durations.

**Figure 18:**
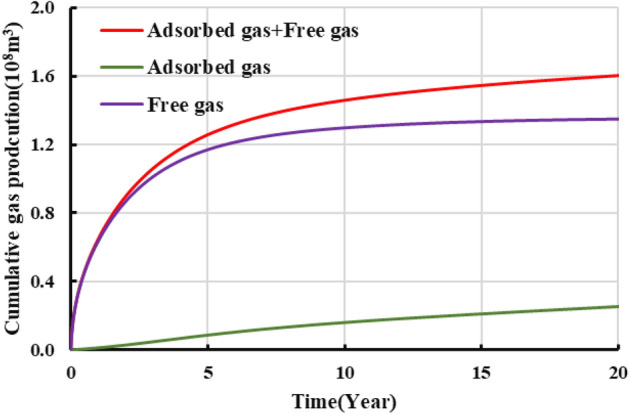
Variation in the gas composition with production time.

**Figure 19:**
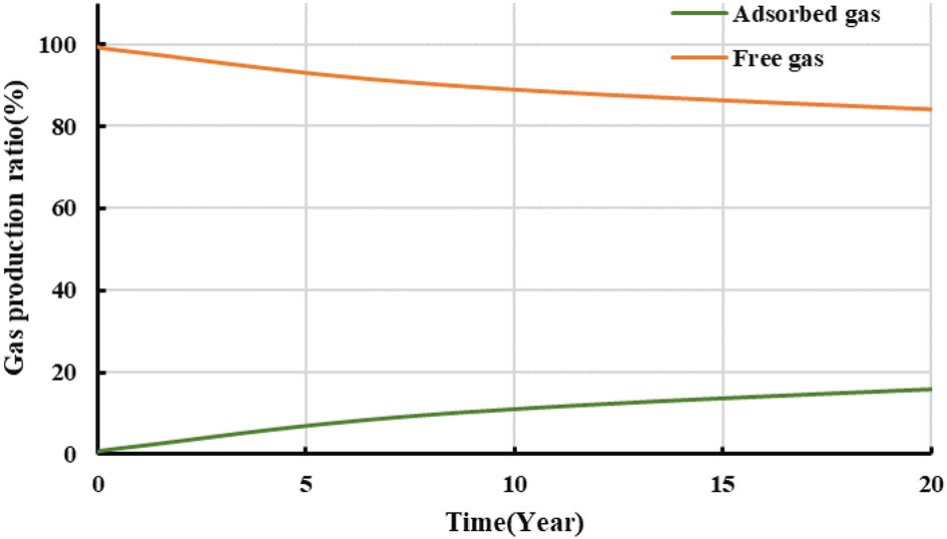
Variation in the proportions of adsorbed and free gas with production time.

The developed productivity model was used to investigate well NH3-5–1 in terms of development dynamics and the variation in adsorbed and free gas production over a 20-year-period. Figure 18 shows the results. For the initial stage of production (i.e., approximately the first two years of production), the cumulative gas production curve basically overlaps the free gas production curve, while the adsorbed gas production is relatively low. Therefore, free gas accounts for the majority of the gas produced during the initial stage. For the middle and later stages of production, the cumulative gas production curve gradually deviates from the free gas production curve, while the adsorbed gas production increases appreciably. The EUR of the gas well is 156 million m^3^, of which adsorbed and free gas account for 34 and 122 million m^3^, respectively. Our observation of Fig. 19 shows that the proportion of adsorbed gas increases gradually as production proceeds, which differs completely from the pattern of variation in the proportion of free gas. Ultimately, adsorbed gas accounts for as much as 20% of the total gas produced from the well. Analysis of the variation in the gas production yielded by the model with the formation pressure reveals the following. At an average formation pressure greater than 35 MPa, the cumulative gas production is basically linearly related with the apparent formation pressure. The slope of the corresponding straight line is 1.81 million m^3^/MPa. Under this condition, the cumulative gas production curve also basically coincides with the free gas production curve, suggesting that the gas produced from the well is basically in the free state. As the formation pressure decreases below 35 MPa, the adsorbed gas begins to be extracted and contributes more to gas production. Consequently, the cumulative gas production curve begins to deviate from the straight line and gradually bends upward. As the apparent formation pressure decreases, the extent to which each of the adsorbed and cumulative gas production curves bends upwards increases. The average slopes of these two curves are 3.6 and 4.02 million m^3^/MPa. The adsorbed gas content of the gas produced from the well plays a relatively crucial role. At the final stage of production (i.e., at a reservoir pressure of approximately 10 MPa), the cumulative adsorbed gas production reaches 340 million m^3^, accounting for approximately 21.79% of the EUR. Therefore, the role of adsorbed gas in the dynamic production process becomes increasingly prominent during the later stage when the reservoir pressure is relatively low. The productivity of the reservoir relies predominantly on the desorption of adsorbed gas during the later stage.

**Figure 20:**
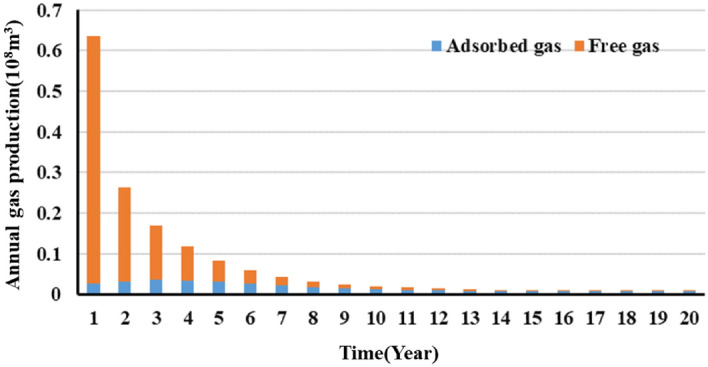
Variation in the annual gas production calculated for the well with production time.

**Figure 21:**
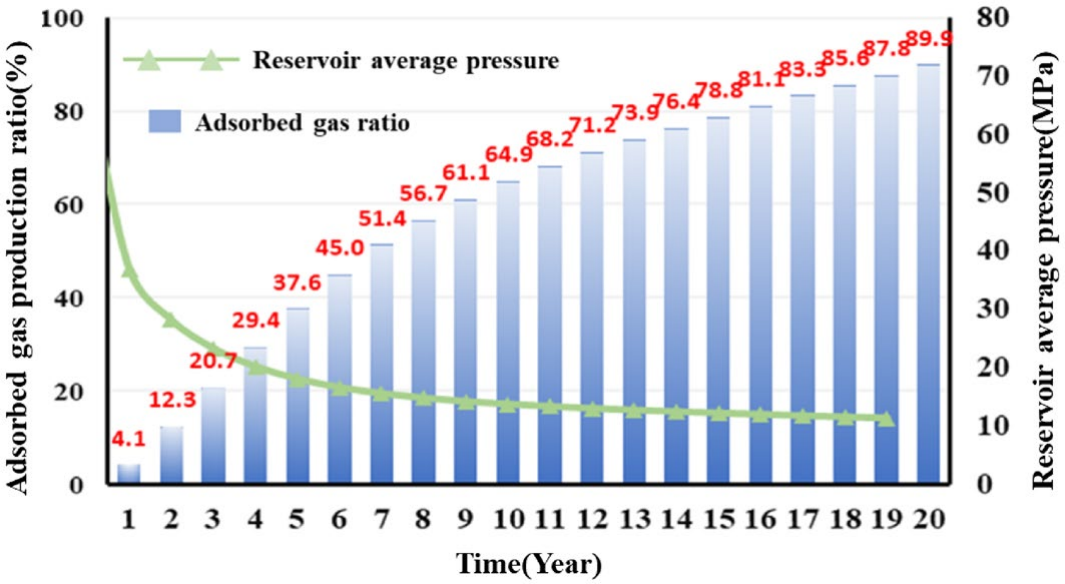
Variation in the annual contribution rate of adsorbed gas and the average pressure in the reservoir with production stage (time).

Observation of Figs. 20 and 21 shows the following. During the first year of production of the gas well, the pressure in the reservoir averages approximately 40 MPa. Free gas accounts for the majority of the produced gas, while adsorbed gas remains basically unextracted and contributes only 4.1% of the total annual gas production. In addition, 40.8% of the EUR is recovered during the first year. Over time, the pressure in the reservoir decreases, and the annual gas production decreases in a notably L-shaped pattern. Free gas is extracted in large quantities, while the adsorbed gas production increases gradually. In the seventh year, adsorbed gas accounts for as much as 50% of the produced gas, while the pressure in the reservoir decreases below 20 MPa, with more than 80% of the EUR having been recovered. During the middle and later stages, annual gas production remains at a constant low rate of 100 million m^3^, the majority of which is supplied by the extraction of adsorbed gas. In the 20th year, the remaining formation pressure averages 10 MPa, an approximately 85% decrease compared with its initial level. The gas production is calculated to be 155.6 million m^3^. The cumulative amount of adsorbed gas produced from the well makes up 21% of the EUR. Therefore, the potential of adsorbed gas should be fully exploited during actual production to improve daily and cumulative gas production and ensure a long period of stable production from the gas well.

## Discussions

This paper designed a full-diameter shale core development physical simulation under high pressure conditions for over 1000 days, taking place of the small shale core short term extraction experiments in previous studies, which makes it reliable to simulate field-scale shale gas production. However, high temperature condition was not included in the proposed experiment in the paper, which is a future research direction in physical simulation of high pressure-high temperature gas extraction for shale gas reservoirs.

In addition, the proposed model is incorporated in comprehensive gas nonlinear effects, including its physical properties at high pressures, supercritical desorption characteristics, and multiple flow mechanisms. This model is more convincing in predicting and analyzing shale gas production decline trend in different production duration and produced gas compositions. For instance, when the non-linear gas flow mechanism is not considered in the production model, the predicted 20-year-production can be underestimated by 11.3%; the calculated EUR of considering stress sensitivity is 7.5% lower than that model predictions without involving stress sensitivity. Likewise, the model considering the high-pressure adsorption predicts that EUR is 1.87% higher than that of only considering Langmuir adsorption. If the above comprehensive factors are considered in the model, the EUR is about 2.5% higher than that of the linear production model. For that reason, the more comprehensive factors considered in the production model is conducive to enhance the accuracy of predicting the medium and long-term productivity. It aims to provide reliable reference to accurately estimate shale gas well production at different production durations.

However, the proposed production model is related to only describe single gas flow process, without considering the gas–water two phases transport in the backflow period. The gas–water flow characterization is exactly to be involved in the production model in the future works.

To sum up, the findings from study can help us explicitly understand and predict shale gas process out of subsurface formations under high geological pressure conditions, dominated by multiple flow mechanisms.

## Conclusions

In this study, a 3343-day ultralong-production-cycle development simulation was conducted with methane as the test gas. As indicated by the pressure and gas production rate curve, the obtained gas accumulation and production characteristics were highly consistent with those observations in the reservoir. During gas-well production, the low gas flowability in the shale matrix led to relatively slow pressure propagation. As a result, only 71% of the EUR was recovered after 3343 days. The production decreased rapidly during the initial stage, followed by a long period of low and stable production during the later stage. This phenomenon was basically consistent with that inreal gas well. A critical desorption pressure (approximately 12 MPa) was found for the extraction of adsorbed gas. Adsorbed gas did not contribute to gas production until the pressure decreased below the critical desorption pressure.

A five-region seepage model that accounts for high-pressure supercritical adsorption, apparent permeability, and stress sensitivity was developed and subsequently validated based on the physical simulation test results. Then it was proved that more gas flow nonlinear effects considered in the proposed model can effectively promote higher prediction accuracy in the medium and long-term productivity of the gas well.

In addition, the production decline analysis were conducted on the specifically single well production data with the production model, which illustrated that free gas accounts for the majority of the produced gas during the initial extraction stage. In the first year, 40.8% of the EUR is recovered, with free gas accounting for more than 90% of the produced gas. During the later production stage, the adsorbed gas is a primary source of gas. Adsorbed gas contributes as much as 50% of the total gas produced in the seventh year. The 20-year-cumulative adsorbed gas production makes up 21% of the EUR. Adsorbed gas is a major factor that can ensure a long period of stable production during the later stage.

During the later stage of production, adsorbed gas begins to desorb, resulting in expanded pore flow channels and an increase in gas flowability. The decreased pressure also enhances gas diffusion and thereby correspondingly increases gas flowability. Therefore, shale gas should be extracted from a reservoir by means of depressurization. The bottomhole pressure should be decreased below the critical desorption pressure as fast as possible to enable effective extraction of the adsorbed gas. However, in terms of the gas-well development, gas should be produced at a controlled pressure for a certain period of time during the initial stage to reduce the negative impact of fracturing fluid flowback on the production, fracture and fracture network closure, and sand production. Subsequently, the pressure should be reduced as fast as possible to exploit the adsorbed gas, aimed at improving daily gas production and ultimate recovery.

## Data Availability

The datasets generated and/or analyzed during the current study are not publicly available due to the fact that the data forms part of current ongoing project but are available from the corresponding author on reasonable request.
